# Microbiome analysis of raw honey reveals important factors influencing the bacterial and fungal communities

**DOI:** 10.3389/fmicb.2022.1099522

**Published:** 2023-01-12

**Authors:** Zirui Ray Xiong, Jonathan H. Sogin, Randy W. Worobo

**Affiliations:** Department of Food Science, Cornell University, Ithaca, NY, United States

**Keywords:** honey, microbiome, metabarcoding, physicochemical properties, alpha diversity and beta diversity

## Abstract

Raw honeys contain diverse microbial communities. Previous studies have focused on isolating bacteria and fungi that are culturable, while missing a large proportion of the microbial community due to culture-based constraints. This study utilized next-generation sequencing (NGS) to analyze the composition of microorganisms in raw honey; these data can reveal environmental and physicochemical variables that are associated with different microbial communities. To examine the microbial composition (bacteria and fungi) of raw honey and analyze its association with physicochemical properties, four types of honey (monofloral, wildflower, manuka, and feral; *n*_total_ = 36) were analyzed *via* amplicon metagenomics. The analyzed honey samples had relatively similar bacterial communities but more distinct and diverse fungal communities. Honey type was determined as a significant factor influencing alpha and beta diversity metrics of bacterial and fungal communities. For the bacterial communities, titratable acidity (TA) was associated with community richness and diversity. For the fungal communities, Brix, TA, and color were associated with community richness, while water activity and color were associated with community diversity. Additionally, important bacterial and fungal amplicon sequence variants (ASVs) that influenced the overall community were identified. Results from this study provide important insights into the microbial communities associated with different types of raw honey, which could improve our understanding of microbial dynamics in beehives, improve honey production, and prevent honeybee disease.

## 1. Introduction

Honey has a diverse microbiome, most of which originates from pollen, flowers, soil, air, dust, and the honeybee digestive tract (Snowdon and Cliver, 1996). Additionally, some secondary microbial contaminants may be introduced into honey during human processing (Snowdon and Cliver, 1996; Olaitan et al., 2007). Honey has a water activity between 0.50 and 0.65. It is generally acidic, with pH ranging from 3 to 5 due to the presence of organic acids like gluconic acid (Olaitan et al., 2007; Balzan et al., 2020). The physicochemical properties of honey have an influence on the microbial communities. The low water activity, low pH, and antimicrobial components (including hydrogen peroxide, antioxidants, and antimicrobial peptides) of honey inhibit the growth of vegetative bacterial cells (Olaitan et al., 2007). Few organisms can survive the osmotic stress of honey; those that do are mainly spore-forming bacteria and yeasts. Previous studies found osmotolerant bacteria that were transmitted to honey from flower nectar through bee pollination (Álvarez-Pérez et al., 2012; Fridman et al., 2012).

Honey-associated microorganisms can be grouped into three types based on origin and ecological niche: bee gut microorganisms, plant-associated microorganisms, and bee pathogens (Bovo et al., 2018). *Lactobacillus* and *Bifidobacterium* lactic acid bacteria (LAB) are major components of the bee gut microbiome and are relatively conserved in honeybee digestive tracts globally (Anderson et al., 2013; Raymann and Moran, 2018). These genera have been found in bee-collected nectar and honey (Olofsson and Vásquez, 2008). Up to 10^8^ CFU per gram of viable LAB have been found in different honey samples (Vásquez et al., 2012). A few other bacterial genera are frequently, though not ubiquitously, found in honeybee digestive tracts; these include *Apibacter*, *Acetobacter*, and *Asaia* (Raymann and Moran, 2018). Some rarer bacteria that can cause disease in and death of honeybees may also be found in honeybee digestive tracts; these include *Enterobacter*, *Klebsiella*, *Citrobacter*, and *Serratia* (Raymann and Moran, 2018). Fungal genera found in honeybee digestive tracts include *Saccharomyces*, *Zygosaccharomyces*, and *Candida* (Yun et al., 2018).

Bacteria and fungi that are commonly found in plants and soil can be transmitted to beehives through pollination. These plant-associated microorganisms are present in honey and other bee products like bee bread (a fermented mixture of pollen and nectar used as food for bees), and some of these microorganisms are beneficial to the bee colonies (Kurek-Górecka et al., 2020). One example is *Actinobacteria*. Even though some *Actinobacteria* spp. are plant pathogens, many of them are protective microbes for honeybees and other insects because they produce secondary metabolites which prevent fungal growth and spoilage (Mohr and Tebbe, 2006; Barke et al., 2010; Anderson et al., 2013). A variety of *Enterobacteriaceae* and *Firmicutes* were found in flowers including *Lactobacillus*, *Bacillus*, and *Weissella* spp., many of which are present in honeybee digestive tracts and honey products. *Lactobacillus kunkeei* has been found in flowers, honeybee gut, and bee bread (Anderson et al., 2013). The ubiquitous presence of LAB across different bee species is the result of horizontal transmission between beehives and environment. The high similarity between *Firmicutes* found in flower nectar and those isolated from honeybee hives is further indication that horizontal transmission of these bacteria happens through pollination (Vásquez et al., 2012). The plant-associated bacteria *Paenibacillus* spp. are commonly found in soil. Some species of *Paenibacillus* are bee pathogens: *P. larvae* is the causative agent for American foulbrood disease; *P. alvei* is commonly found as a secondary invader of European foulbrood disease caused by *Melissococcus plutonius* (Genersch, 2010). As for the common plant-associated fungi found in honey, *Cladosporium* is a filamentous fungus that is common in the environment, and some species are potential plant pathogens (Bensch et al., 2012). It was proposed that *Cladosporium* could cohabit with bees and transmit from plant or bees to persist in bee products (Martinson E. O. et al., 2012). Other filamentous fungi that are commonly found in plant pollen include *Botrytis*, *Penicillium*, and *Mucor*, which are transmitted to honeybees and frequently found in bee bread (Disayathanoowat et al., 2020). Some common genera of yeast that were isolated from pollen and bee bread include *Candida*, *Cryptococcus*, *Kloeckera*, *Metschnikowia*, and *Rhodotorula* (Gilliam et al., 1974). Flower-derived microorganisms are subjected to environmental changes, which, in turn, contribute to the variation, growth, and secondary metabolite production of other environment-derived microorganisms in honeybees (Vásquez et al., 2012).

Overall honeybee health can be threatened by bacterial and fungal pathogens, which may contribute to colony collapses. Common bacterial pathogens include *Melissococcus*, *Paenibacillus*, and *Spiroplasma*; Fungal pathogens for honeybees include *Ascosphaera*, *Aspergillus*, and *Nosema* (Schwarz et al., 2015). As a common mold found in the environment, *Aspergillus* is an opportunistic pathogen that can infect honeybee larvae and cause stonebrood disease. The common chalkbrood disease is caused by *Ascosphaera apis*, while nosema disease is caused by spore-forming fungi *Nosema apis* and *Nosema ceranae* (Jensen et al., 2013; Schwarz et al., 2015).

As previous studies suggest, microbial and honeybee DNA present in honey reflect the hive microbiome and honeybee hologenome; these data may reveal the bee pathosphere and indicate overall bee colony health (Hamdi et al., 2011; Engel et al., 2016; Raymann and Moran, 2018; Bovo et al., 2020). Analyzing the honey microbiome can potentially help in the understanding of microbial hive dynamics, which may improve honey production and prevent honeybee diseases. However, most previous honey microbiome studies use traditional culture-based methods to isolate and identify microorganisms in honey, which is subject to culture biases (Anderson et al., 2013). Using culture-independent methods to investigate the microbiome of honey avoids biases induced by researcher-selected growth conditions. Recent studies have used next-generation sequencing (NGS) methods to study the microbiome of honeybee gastrointestinal tracts, pollen, and bee bread, while metagenomic analyses of honey are limited (Engel et al., 2012; Moran et al., 2012; Powell et al., 2014; Jones et al., 2018; Yun et al., 2018; Disayathanoowat et al., 2020).

In our study, we used 16S rRNA and internal transcribed spacer (ITS) gene metabarcoding method to evaluate and compare the microbiomes of raw honey derived from different sources. We selected two common types of honey (monofloral and wildflower) from central NY region. Monofloral honey is predominantly from the nectar of a single plant species, while wildflower honey is produced from multiple plant species (Alvarez-Suarez et al., 2014). To compare the differences between different honey types, we also chose to include two special types, manuka honey and feral honey that have not been studied previously and could potentially have distinct and interesting microbial communities. Manuka honey is a highly valuable New Zealand monofloral honey with antimicrobial and antioxidant capabilities (Niaz et al., 2017). The high content of antioxidants could be produced by certain microorganisms in honey, which would deter the growth of other microorganisms in the environmental niche (Brudzynski, 2021). Feral honey is produced by domesticated western honey bees *Apis mellifera* that swarmed and established wild colonies (Hinshaw et al., 2021). Feral colonies are able to survive in the wild without human management and develop mechanisms to defend against varroa mites and other pathogens (Youngsteadt et al., 2015). The microbiome of feral honeybees is potentially associated with the strong immune systems and mite survival strategies of these bee colonies, which could potentially be reflected in the honey.

Previous studies have reported the association between honey microbiomes and parameters like moisture, electrical conductivity, and botanical origin (Wen et al., 2017; Balzan et al., 2020; Kňazovická et al., 2020). To further evaluate different physicochemical parameters of raw honey and their association with the microbiome of different types of honey, we measured honey pH, water activity, Brix, titratable acidity, and color and evaluated their association with microbial community diversity. We found that the bacterial communities among honey samples were relatively conservative, while fungal communities were more diverse. Some physicochemical properties of honey, including titratable acidity, water activity, and color, were associated with microbiome composition. To the best of our knowledge, this is one of the first articles assessing the microbiome of manuka honey and feral honey *via* amplicon metagenomics.

## 2. Materials and methods

### 2.1. Honey sample collection

In this study, we performed physicochemical and microbiome analysis on four types of honey: monofloral, wildflower, manuka, and feral. Monofloral honey was purchased from two local honey shops (Ithaca, NY, United States). To explore the diversity in their microbial composition, monofloral honeys from different floral sources were selected, including basswood, bamboo, buckwheat, orange blossom, goldenrod, and black locust. Wildflower honey was purchased from three honey shops in the central NY region. For the New Zealand manuka honey, three different brands were purchased online. Three feral honey samples were provided by a local beekeeper (Utica, NY), where the honeys were collected from swarmed honeybees. Honey samples were stored at room temperature until processing, due to honey’s shelf-stable nature. A total number of 36 honey samples were analyzed in this study.

### 2.2. Physicochemical analysis of raw honey

All four types of honey samples (seven monofloral, five wildflower, three manuka, and three feral) were subject to physicochemical analysis. pH, titratable acidity, and Brix were measured using pH meter (pHi 470, Beckman Coulter, Brea, CA, United States), automatic titrator (Ti-Note EasyPlus Titrators AP002, Mettler Toledo, Columbus, OH, United States), and pocket digital refractometer (Sper Scientific, Scottsdale, AZ, United States). Water activity was measured with water activity meter (AQUALAB 4TE, METER Group, Pullman, WA, United States) and color was measured with Chroma Meter (Konica Minolta CR-400, Tokyo, Japan) using CIELAB scale. All measurements were performed in triplicate.

### 2.3. DNA extraction, library preparation, and illumina amplicon sequencing

Each honey was sampled twice and treated as biological duplicates for genome extraction. Honey was dissolved in phosphate-buffered saline (PBS) and treated with 1500 U/ml catalase to remove hydrogen peroxide that could be produced during dilution (Brudzynski et al., 2011; Chen et al., 2012). The 50% (w/w) honey solution was incubated at room temperature for 2 h and centrifuged at 10,000 rpm, 4°C for 15 min. The pellet was resuspended in 10 ml PBS and centrifuged at 10,000 rpm, 4°C for another 15 min. DNA was extracted from this pellet with the DNeasy PowerSoil Pro Kit according to the manufacturer’s recommendation. Illumina MiSeq library preparation for 16S rRNA and ITS gene amplicon was performed. The 16S V3-V4 region was amplified with primers IL_Bakt341F (CCTACGGGNGGCWGCAG) and IL_Bakt805R (GACTACHVGGGTATCTAATCC; Herlemann et al., 2011; Klindworth et al., 2013). A 0–4 bp heterogeneity spacer between Illumina index sequence and the 16S locus-specific primer was included to improve sequencing quality on the flow cell (Fadrosh et al., 2014). The ITS 5.8S-ITS2 region was amplified with primer IL_5.8SFungF (AACTTTYRRCAAYGGATCWCT) and IL_ITS4FungR (AGCCTCCGCTTATTGATATGCTTAART; Taylor et al., 2016). Similarly, a 0–4 bp heterogeneity space was added between Illumina index sequence and the ITS primer. A two-step library preparation was adapted from a previous study by Holm et al. (2019). Successful target amplification from the first PCR was verified by gel electrophoresis and samples were then submitted to the Cornell Biotechnology Resource Center, Cornell Institute of Biotechnology (Ithaca, NY), for indexing and sequencing. Samples were quantified by Qubit 4 Fluorometer and then normalized prior to performing unique dual indexing. After dual indexing, samples were pooled and the library was cleaned using AMPure XP beads. Quality control with fragment analysis confirmed the correct distribution of fragment lengths. An Illumina MiSeq 2 × 250 bp (V2 chemistry) reagent kit was used to sequence the library. Two PCR negative controls (no DNA template for PCR reaction) and four extraction negative controls (no honey sample for DNA extraction) were included in this study. A total number of 84 amplicon samples were sequenced.

### 2.4. Data analysis

QIIME 22021.11.0 was used to process and analyze the demultiplexed 16S and ITS amplicon sequencing data (Bolyen et al., 2019). Primers were trimmed from raw reads of 16S and ITS sequences using q2-cutadapt plugin. To achieve more accurate fungal taxonomic classification, demultiplexed ITS sequences were trimmed and conserved regions were removed using the q2-ITSxpress plugin (Rivers et al., 2018). DADA2 was used to filter, denoise, and merge trimmed reads to identify all observed amplicon sequence variants (ASVs; Callahan et al., 2016). The chimeric sequences identified by DADA2 were removed. Taxonomy assignment was performed using a precomputed naïve Bayesian classifier (SILVA version 138 reference alignment for 16S rDNA sequences and UNITED version 8.3 database for ITS sequences) using q2-feature-classifier (Bokulich et al., 2018).

Downstream analyses and visualization, including diversity analysis, statistical testing, and microbial community composition were performed in R (version 4.1.1). For 16S and ITS sequences, ASVs identified as mitochondria or chloroplast by the classifier were treated as contaminants and removed. Unknown ASVs at the phylum level were removed; these were typically unassigned mitochondria or chloroplast sequences (data not shown). Frequency- and prevalence-based *de novo* classification methods were used to identify extraction and PCR contaminants using “decontam” package in R (Davis et al., 2018). ASVs that were present predominantly in extraction negative controls and PCR negative controls were removed from true samples. Sequences were rarefied and normalized with “phyloseq” package in R by resampling the abundance values to achieve parity between samples (McMurdie and Holmes, 2013). The most abundant bacterial and fungal ASVs in honey samples were visualized with “ggpubr” package (version 0.4.0). Alpha diversity metrics, including Shannon diversity, Simpson and inverse Simpson diversity, Pielou’s evenness, ACE, and Chao richness metrics, were calculated with “vegan” R package (version 2.5-7). For normally distributed alpha diversity metrics, ANOVA with Tukey’s honest significance test was used to perform pairwise comparisons between groups of categorical variables. General linear model with normal distribution was used to fit alpha diversity metrics to continuous variables. For non-normally distributed alpha diversity metrics, Wilcoxon rank sum test with false discovery rate (FDR) corrections for multiple comparisons. Kruskal–Wallis tests were performed on categorical variables. Generalized linear model with quasi-Poisson distribution was used to fit continuous variables. For beta diversity, Bray–Curtis dissimilarity, Jaccard distance, and phylogeny-based UniFrac (weighted and unweighted) metrics were calculated (Jaccard, 1912; Beals, 1984; Lozupone and Knight, 2005; Lozupone et al., 2007). To visualize the differences in microbiome composition, beta diversity metrics were plotted with non-metric multidimensional scaling (NMDS). The multivariate homogeneity of group dispersion was tested by beta dispersion and the community composition was compared with permutation analysis of variance (PERMANOVA) using the “adonis” function in the “vegan” package with 1,000 permutations. Additional visualizations, including heat maps and Venn diagrams, were created in R.

## 3. Results

### 3.1. Honey physicochemical properties

The physicochemical properties of honey samples, including Brix, pH, titratable acidity, color, and water activity, are summarized in Table 1. Feral honey sample FF1/2 was missing physicochemical data due to limited sample quantity. For the same reason, FF1/2 was subjected to 16S rRNA and ITS amplicon sequencing but was removed when performing diversity analyses.

**Table 1:** Physicochemical properties of honey.

Sample ID	Honey type	Brix	pH	TA	Color_L*	Color_a*	Color_b*	Aw
S1/2	Manuka	77.33 ± 0.76	3.91 ± 0.05	0.03427 ± 0.00652	29.67 ± 0.43	2.26 ± 0.21	10.62 ± 0.89	0.6087 ± 0.0072
NZ1/2	Manuka	77.93 ± 0.90	4.08 ± 0.10	0.02843 ± 0.00590	30.45 ± 0.72	0.94 ± 0.04	11.64 ± 0.53	0.5985 ± 0.0035
A1/2	Manuka	76.03 ± 1.24	3.74 ± 0.03	0.03327 ± 0.00580	30.72 ± 0.34	2.23 ± 0.21	12.42 ± 0.15	0.6002 ± 0.0015
HRB1/2	Monofloral	81.77 ± 0.15	4.06 ± 0.02	0.03222 ± 0.00498	32.77 ± 1.10	0.16 ± 0.02	9.25 ± 0.33	0.5433 ± 0.0032
HRO1/2	Monofloral	79.2 ± 2.86	3.69 ± 0.05	0.03465 ± 0.00322	31.56 ± 0.02	0.37 ± 0	11.85 ± 0.28	0.5285 ± 0.0096
HRG1/2	Monofloral	78.3 ± 3.14	4.07 ± 0.04	0.03442 ± 0.00553	30.79 ± 0.49	1.06 ± 0.12	12.34 ± 0.25	0.5425 ± 0.0029
WBB1/2	Monofloral	79.73 ± 2.40	4.05 ± 0.02	0.01967 ± 0.00413	29.1 ± 0.55	3.59 ± 0.07	12.75 ± 0.24	0.5465 ± 0.0055
WBW1/2	Monofloral	80.37 ± 0.93	4.2 ± 0.17	0.03327 ± 0.01038	32.8 ± 0.60	−0.01 ± 0.07	8.51 ± 0.41	0.5487 ± 0.0032
WBU1/2	Monofloral	79.63 ± 1.70	3.79 ± 0.06	0.03756 ± 0.00495	22.65 ± 0.31	3.91 ± 0.04	3.8 ± 0.06	0.5602 ± 0.0010
WL1/2	Monofloral	78.73 ± 2.85	4.11 ± 0.03	0.02223 ± 0.00417	32.8 ± 0.16	0.09 ± 0.05	8.84 ± 0.13	0.5226 ± 0.0050
W20F1/2	Wildflower	81.8 ± 0.26	4.23 ± 0.17	0.01302 ± 0.00179	29.04 ± 0.30	2.78 ± 0.09	12.22 ± 0.11	0.561 ± 0.0030
W21S1/2	Wildflower	82.27 ± 0.15	4.3 ± 0.17	0.01152 ± 0.00439	30.84 ± 0.21	0.45 ± 0.09	10.87 ± 0.29	0.5378 ± 0.0039
W21F1/2	Wildflower	80.03 ± 0.60	3.93 ± 0.08	0.01317 ± 0.00312	32.86 ± 0.14	−1 ± 0.02	7.38 ± 0.08	0.5761 ± 0.0029
JS1/2	Wildflower	77.77 ± 0.35	3.75 ± 0.06	0.01245 ± 0.00528	41.05 ± 0.36	−1.04 ± 0.02	7.84 ± 0.19	0.5651 ± 0.0041
KH1/2	Wildflower	82.8 ± 0.46	4.05 ± 0.08	0.01352 ± 0.00180	32.14 ± 0.50	0.84 ± 0.06	11.35 ± 0.17	0.5287 ± 0.0034
FR1/2	Feral	77.4 ± 0.17	4.29 ± 0.09	0.0119 ± 0.00230	29.6 ± 0.45	0.16 ± 0.05	3.21 ± 0.04	0.6337 ± 0.0018
FF1/2	Feral	NA*	NA	NA	NA	NA	NA	NA
FH1/2	Feral	77.03 ± 0.15	3.84 ± 0.04	0.0208 ± 0.00133	35.64 ± 0.88	−0.62 ± 0.01	8.1 ± 0.54	0.6168 ± 0.0027

The mean of three measurements and standard deviation are listed.

* Physicochemical data were not available for FF1/2 due to limited amount of honey samples collected.

### 3.2. Amplicon metagenomic sequencing data summary

**Figure 1:**
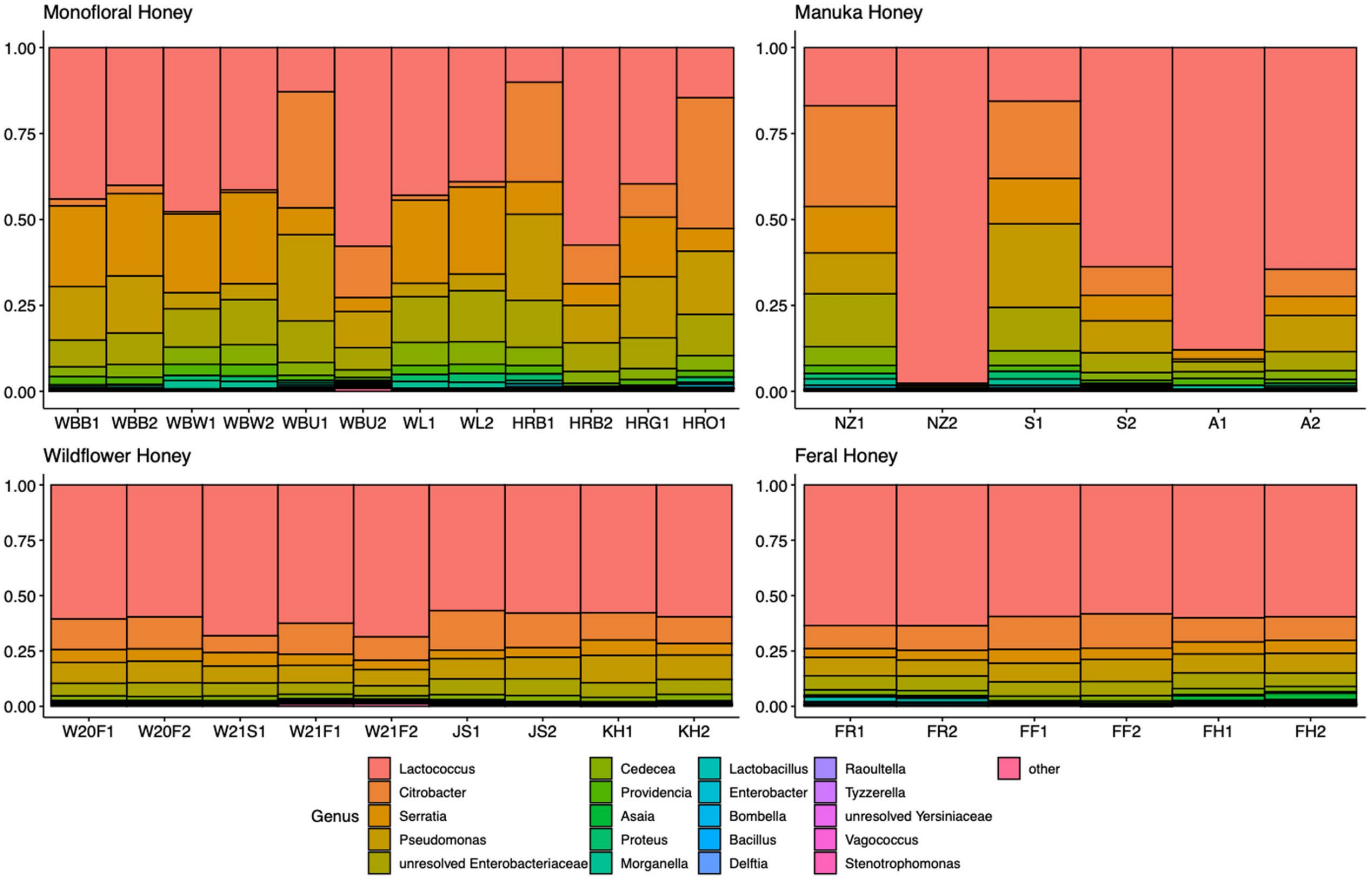
Honey bacterial composition plot. 16S ASVs were agglomerated to the genus level for each honey sample. Genera with relative abundance higher than 0.05% across samples were selected and plotted. Genera with less than 0.05% were agglomerated as “others.”

**Figure 2:**
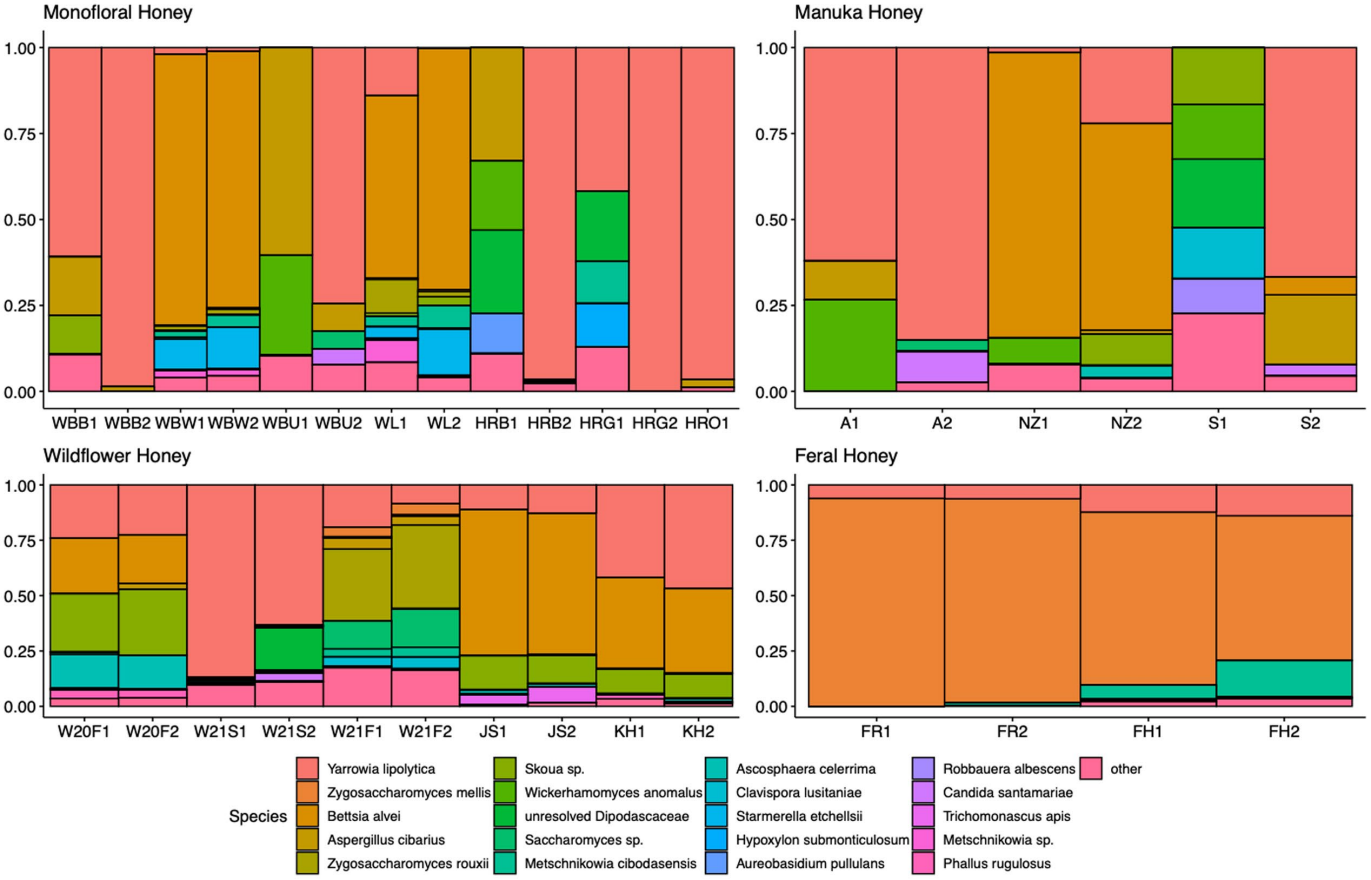
Honey fungal composition plot. ITS ASVs were agglomerated to the species level for each honey sample. Species with relative abundance higher than 0.3% across samples were selected and plotted. Species with less than 0.3% were agglomerated as “others.”

**Figure 3:**
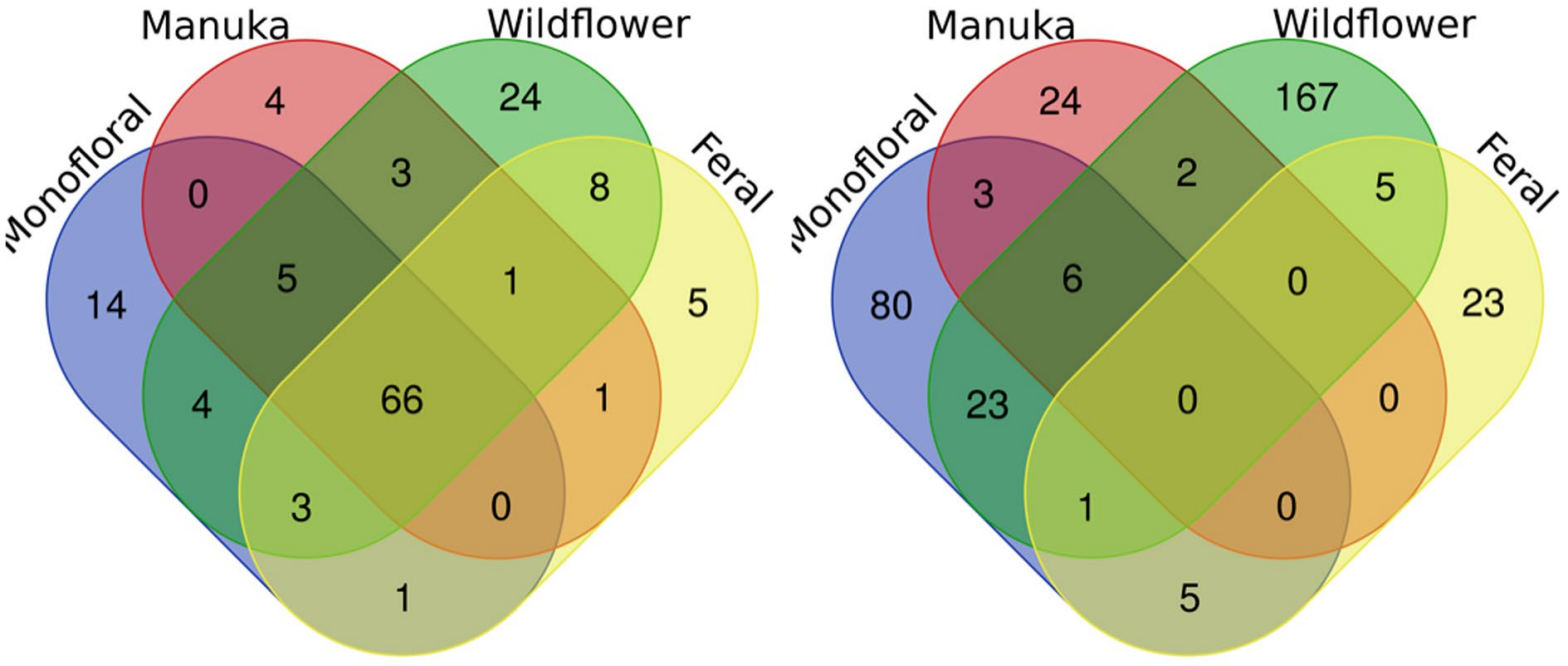
Venn diagrams for bacterial and fungal ASVs of four honey types. Bacterial and fungal ASVs were grouped based on four types of honey: monofloral, manuka, wildflower, and feral. ASVs shared between different types of honey were labeled in the overlapping area in the diagram. Left: bacterial. Right: fungal.

A total of 2,040,648 raw 16S rRNA amplicon reads and 4,084,874 raw ITS amplicon reads were obtained for 42 samples, including four extraction controls and two PCR controls. After trimming adapter sequences and primers, filtering low-quality reads, denoising, and removing chimeric sequences, a total of 1,317,356 16S and 2,308,930 ITS reads remained for downstream analyses. After removing sequences unidentified at phylum level and sequences identified as PCR or extraction contaminants using R package “decontam,” a total number of 1,285,423 16S reads remained. For ITS sequence, there were 2,305,376 reads retained after removing sequences unidentified at class level. After careful consideration, we removed two extraction contaminant ASVs identified as *Yarrowia lipolytica* and only used 830,110 reads of ITS sequence for downstream analysis. Before rarefaction and normalization, samples with low reads (less than 2,000) were removed: 16S sequences of 2 monofloral honey (HRG2 and HRO2) and 1 wildflower honey (W21S2), ITS sequences of 1 monofloral honey (HRO2) and 2 feral honeys (FF1 and FF2). Rarefaction curves for 16S and ITS sequences are visualized in Supplementary Figure 1. All samples reached plateau after resampling, indicating that the sequencing depth was sufficient to capture microbial community diversity of the samples. To visualize the most abundant genera for bacterial composition, bacterial ASVs were agglomerated to the genus level, and genera with abundance higher than 0.05% were selected for honey bacterial composition plot (Figure 1). Similarly, fungal ASVs were agglomerated to the species level, and species with relative abundance higher than 0.3% were selected for honey fungal composition plot to illustrate the most abundant fungal species (Figure 2). The bacterial community showed less variability compared to fungal community, and it was dominated by *Lactococcus lactis*. Some other common genera of bacteria include *Citrobacter*, *Pseudomonas*, *Serratia*, and *Cedecea*. Specific fungal species were dominant in certain samples. *Yarrowia lipolytica* and *Bettsia alvei* were dominant in some of the monofloral, manuka, and wildflower honey, while feral honey was dominated by *Zygosaccharomyces mellis*. Some other fungal species can be found in particular types of wildflower honey, such as *Skoua* sp., *Zygosaccharomyces rouxii*, *Ascosphaera celerrima*, and *Saccharomyces* sp. The core honey microbiome can be represented by shared taxa among different types of honey. As shown in the Venn diagrams (Figure 3), 66 bacterial ASVs were shared among all four types of honey, while there was 0 fungal ASV shared by all four types of honey. Based on our result, we can presume that there is a core bacterial microbiome for honey. Additionally, 167 fungal ASVs were present only in wildflower honey and 80 fungal ASVs were found only in monofloral honey, which further demonstrated that honey has a diverse and distinct fungal community.

### 3.3. Alpha diversity of honey microbial community

**Figure 4:**
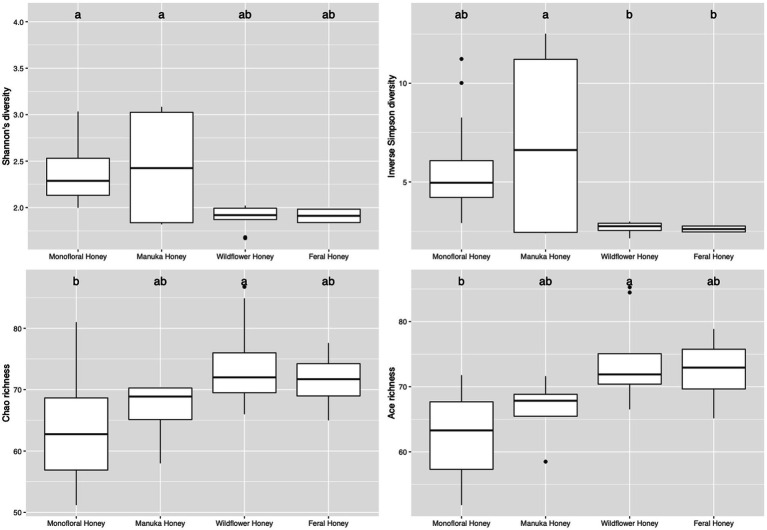
Alpha diversity metrics of honey bacterial community. Diversity was measured with Shannon and inverse Simpson indices. Richness was measured with Chao and ACE indices. Comparison between honey sample types was performed with ANOVA and Tukey’s honest significance test. Letters above the bar plots represented shared significance groups (*p*-value cutoff is 0.05).

**Figure 5:**
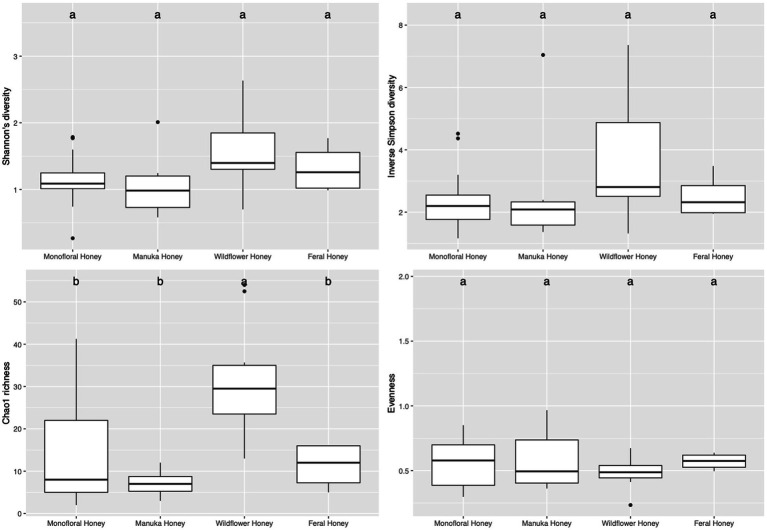
Alpha diversity metrics of honey fungal community. Diversity was measured with Shannon and inverse Simpson indices. Richness was measured with Chao index. Evenness was measured with Pielou’s evenness index. Comparison between honey sample types was performed with ANOVA and Tukey’s honest significance test. Letters above the bar plots represented shared significance groups (*p*-value cutoff is 0.05).

To evaluate the species diversity within each honey type, alpha diversity of bacterial composition was assessed with Shannon diversity, inverse Simpson diversity, Chao richness, and ACE richness indices (Supplementary Table 1), while alpha diversity of the fungal composition was measured with Shannon diversity, inverse Simpson diversity, Chao richness and Pielou’s evenness indices (Supplementary Table 2). Bar plots of alpha diversity indices grouped by honey types were visualized in Figures 4, 5. ANOVA analysis reported *p*-value below 0.05 for all four metrics of bacterial alpha diversity and 1 metric of fungal alpha diversity (Chao richness), indicating that there were differences in the mean of these indices between honey types. Pairwise comparisons using Tukey’s HSD test showed that there were significant differences between the bacterial community richness of monofloral and wildflower honey as measured by Chao and ACE indices (*p* < 0.05). In terms of the bacterial community diversity, there were significant differences between wildflower and monofloral honey as estimated by Shannon diversity metric (*p* = 0.0144) and between wildflower and manuka honey as measured with inverse Simpson diversity (*p* = 0.0416). Considering that Shannon diversity and inverse Simpson diversity metrics were not normally distributed, we thus performed Kruskal–Wallis rank sum test on these two metrics and found that they also differed by honey types (*p* < 0.01). Pairwise comparison was performed using Wilcoxon rank sum exact test and *p*-value was adjusted with false discovery rate (FDR) correction. Monofloral honey was found to be significantly different from both feral and wildflower honey as estimated by Shannon and inverse Simpson diversity metrics (*p* < 0.01). Fungal diversity metrics (Shannon and inverse Simpson) were relatively similar between different honey types. For the fungal community richness, only wildflower honey showed significant difference from the other three types of honey as estimated by Chao richness (*p* < 0.05). Similarly, Kruskal–Wallis rank sum test was used for non-normally distributed Chao richness metric and significant differences were found between groups (*p* < 0.05). Wildflower honey had a significantly different richness compared to the other three types of honey (*p* < 0.05) using Wilcoxon rank sum exact test with FDR adjustment.

Physicochemical properties were tested for their correlations to alpha diversity metrics. For the bacterial community, titratable acidity (TA) was found to be associated with ACE richness by fitting the data to a general linear model (*p*-value = 0.0445). For the non-normal diversity metrics, TA was also found to be significantly correlated with Shannon (*t* value = 4.025, Pr(>|*t*|) = 0.000414, McFadden’s *R*^2^ = 0.375) and inverse Simpson (*t* value = 3.860, Pr(>|*t*|) = 0.000641, McFadden’s *R*^2^ = 0.371) metrics using quasi-Poisson distribution (*p*-value <0.001). Correlations between honey TA and bacterial community alpha diversity metrics (Shannon diversity and inverse Simpson diversity) were visualized in Supplementary Figure 2. For the fungal community composition, Brix, TA, and color (L* and a*) were found to be significantly associated with Chao richness estimator using quasi-Poisson distribution (*p* < 0.05). However, Pielou’s evenness index and diversity metrics were not correlated with any physicochemical properties.

### 3.4. Beta diversity of honey microbial community

**Figure 6:**
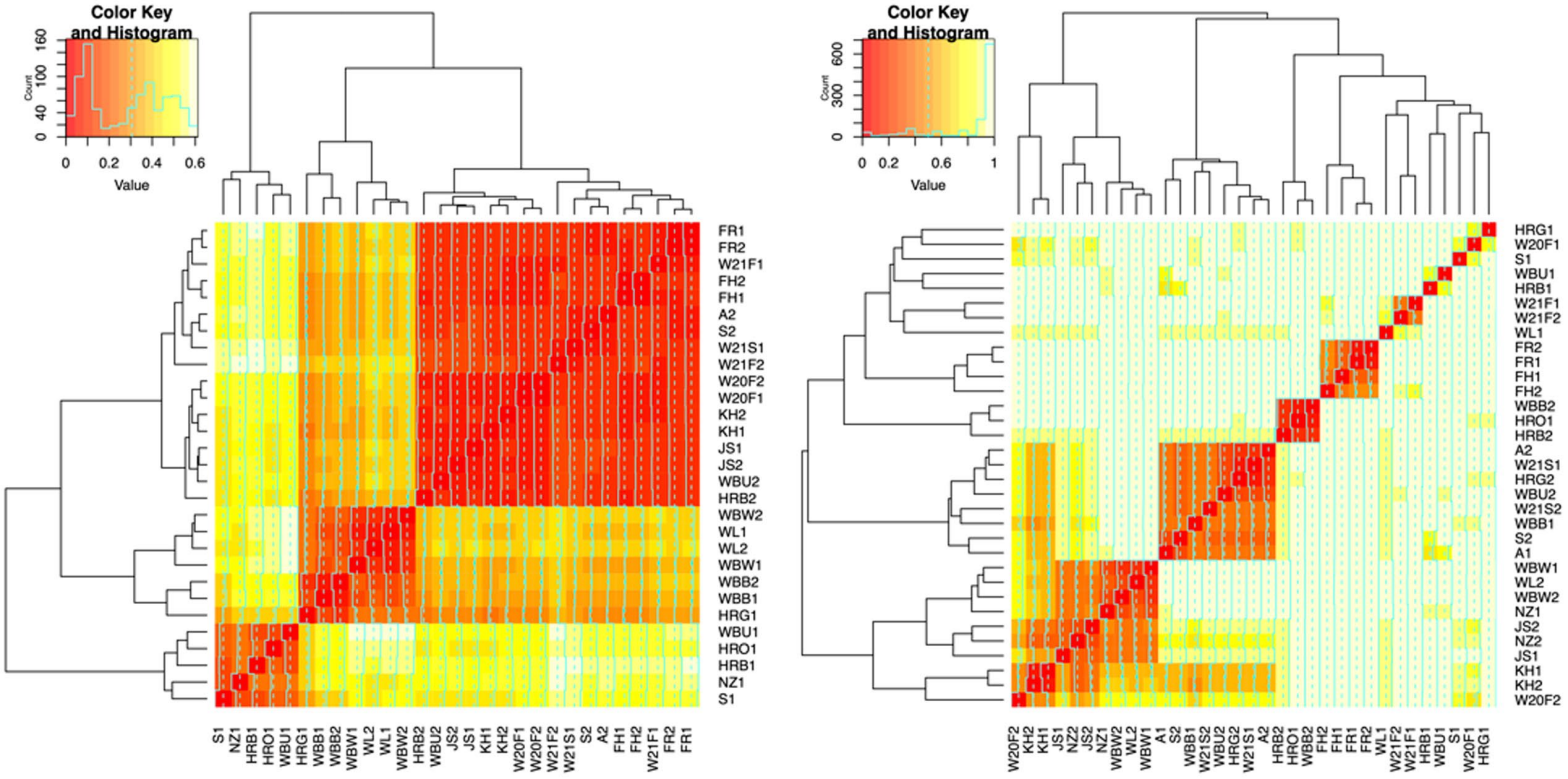
Heat maps of Bray-Curtis distances between honey bacterial and fungal community. Left: bacterial community. Right: fungal community. Each line and column represented a honey sample. The degree of similarity based on Bray–Curtis distances was represented by the color and dendrogram. Color red represented high similarity while light yellow represented low similarity. Samples grouped together in the dendrogram were highly similar. Color key and histogram in the top left corner of the heat map represented the distribution of Bray–Curtis distances. The distance of the line from the center of each color-cell is proportional to the distance value. Axis labels are colored based on honey type: monofloral (black), wildflower (red), manuka (green), feral (blue).

**Figure 7:**
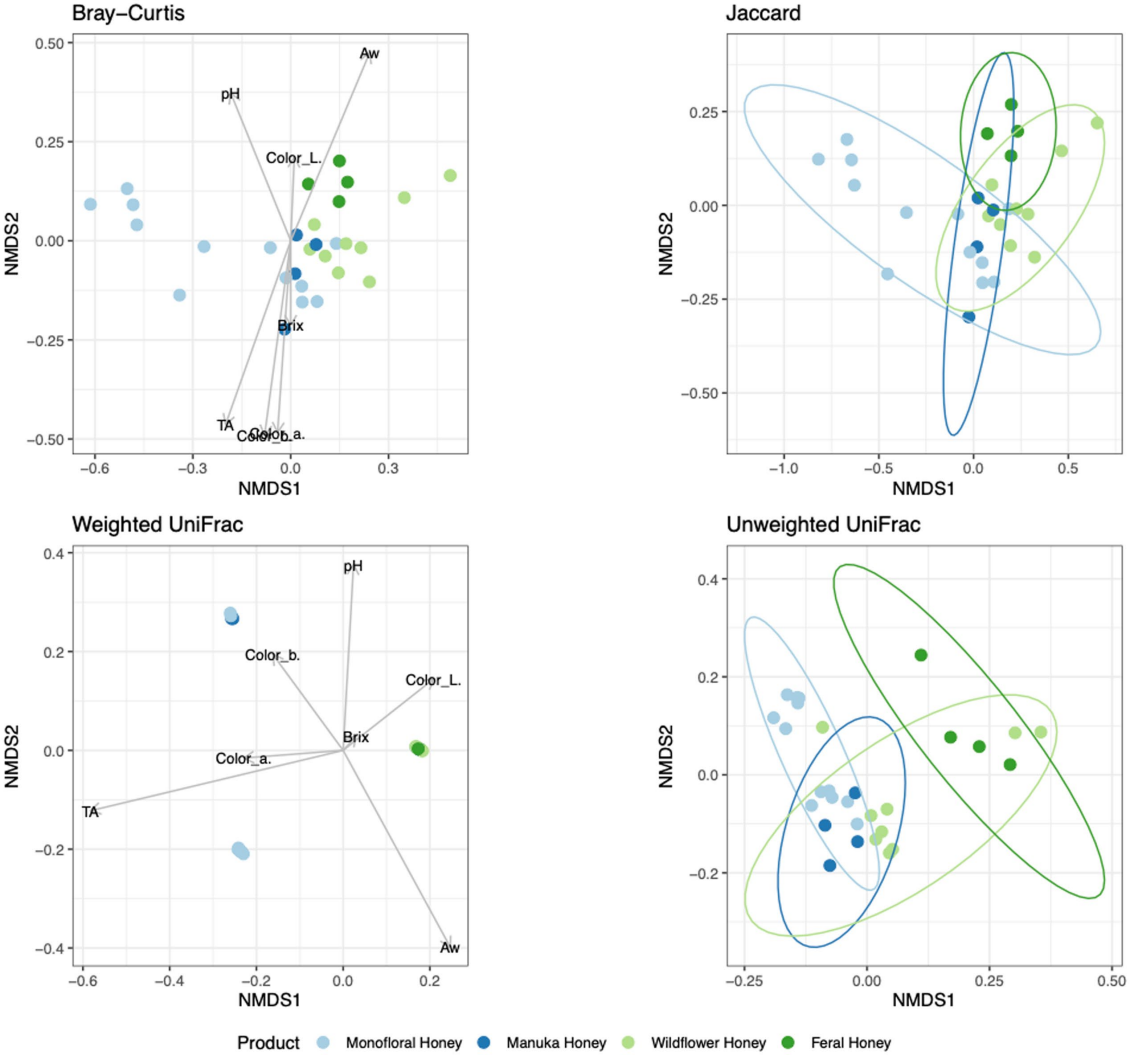
Non-metric multidimensional scaling (NMDS) ordination for bacterial community structure based on the relative abundance of 16S ASVs. Community dissimilarity was evaluated with four metrics: Bray–Curtis, Jaccard, weighted UniFrac, and unweighted UniFrac. Arrowed lines (vectors) showing correlation between physicochemical properties and community dissimilarity were plotted for Bray–Curtis and weighted UniFrac metrics. The vectors represented mean direction and strength of correlation. Ellipses indicating confidence intervals of 95% for all honey types were plotted for Jaccard and unweighted UniFrac metrics.

**Figure 8:**
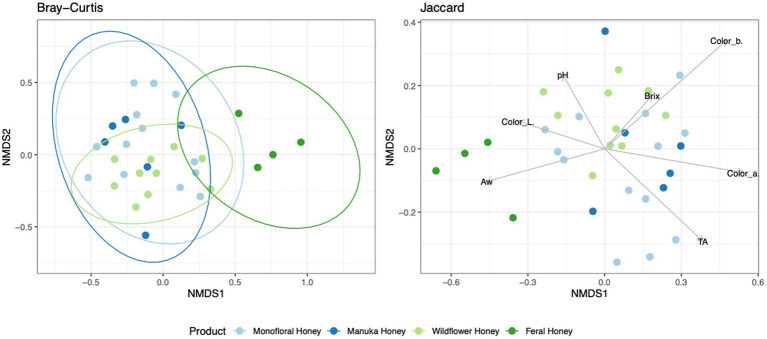
Non-metric multidimensional scaling (NMDS) ordination for fungal community structure based on the relative abundance of ITS ASVs. Community dissimilarity was evaluated with Bray–Curtis and Jaccard metrics. Ellipses indicating confidence intervals of 95% for all honey types were plotted for Bray–Curtis dissimilarity. Arrowed lines (vectors) showing correlation between physicochemical properties and Jaccard dissimilarity were plotted. The vectors represented mean direction and strength of correlation.

To evaluate the degree of differentiation among microbial communities of different honey types, Bray–Curtis and Jaccard beta diversity indices were calculated for both bacterial and fungal community of each sample. Weighted and unweighted UniFrac distance metrics were calculated only for the bacterial community but not for the fungal community, because ITS sequences cannot be used to inform evolutionary distances among distantly related species (Schoch et al., 2012; Lücking et al., 2020). The differences of Bray–Curtis index between samples were visualized with heat map for both bacterial and fungal community (Figure 6). Honey samples were separated into three clusters based on bacterial composition, while fungal composition was divided into 12 clusters. Overall, honey samples used in this study had similar bacterial composition, but the fungal composition was more diverse. Varying degrees of overlap can be observed for clusters of each honey type in NMDS plots, especially for the fungal community of monofloral, wildflower, and manuka honey (Figures 7, 8). PERMANOVA analysis showed significant differences in microbial community composition for different honey types using Bray–Curtis dissimilarity (pseudo *F* = 4.2385, *R*^2^ = 0.33714, *p* = 0.001998 for bacterial community, pseudo *F* = 2.7998, *R*^2^ = 0.22459, *p* = 0.000999 for fungal community). Pairwise comparison between honey types was performed to further evaluate the differences. Results showed significant differences between the bacterial community of monofloral and wildflower honey using Bray–Curtis index (pseudo *F* = 9.0657543, *R*^2^ = 0.32301837, *p* = 0.001, adjusted *p*-value = 0.006). The differences of bacterial community between monofloral and feral honey was also significant (pseudo *F* = 4.3954513, *R*^2^ = 0.23894229, *p* = 0.008, adjusted *p*-value = 0.048). Other distance metrics for bacterial community, including Jaccard, weighted UniFrac, and unweighted UniFrac, showed similar results. As for the fungal community, pairwise comparison between different honey types showed significant differences between monofloral honey and feral honey using Bray–Curtis index (pseudo *F* = 4.8119958, *R*^2^ = 0.24288294, *p* = 0.001, adjusted *p*-value = 0.006). The fungal composition differences between wildflower and feral honey were also significant (pseudo *F* = 7.1475886, *R*^2^ = 0.37328923, *p* = 0.002, adjusted *p*-value = 0.012). Although the adjusted *p*-value was higher than 0.05 for the pairwise PERMANOVA analysis between manuka honey and feral honey using Bray–Curtis distance metric (pseudo *F* = 7.0767424, *R*^2^ = 0.46938140, *p* = 0.009, adjusted *p*-value = 0.054), the difference of Jaccard distance metric for these two honey types was significant, with an adjusted *p*-value lower than 0.05 (*p* = 0.005, adjusted *p*-value = 0.030).

Based on the visualization of the beta diversity metrics and the beta dispersion test, these differences in beta diversity can be attributed to the non-homogeneous distribution of each honey group. Permutation test for homogeneity of multivariate dispersions showed that the group distances of bacterial Bray–Curtis index were significant (*F* = 6.4887, Pr(>*F*) = 0.002997). Pairwise comparison further demonstrated that the dispersion of wildflower and feral honey was significantly different from manuka and monofloral honey (*p* < 0.05). Similarly, the group distances of fungal Bray–Curtis index were significant (*F* = 6.6999, Pr(>*F*) = 0.002997). Further pairwise comparison showed that the beta dispersion of feral honey was significantly different from the other three types of honey (*p* < 0.05).

To elucidate the relationship between physicochemical properties and the microbial community, all physicochemical parameters were treated as continuous variables and fitted to the bacterial Bray–Curtis metric, bacterial weighted UniFrac, and fungal Jaccard metric. Vectors of Brix, pH, TA, water activity, and CIELAB color were visualized in NMDS plots for bacterial Bray–Curtis, bacterial weighted UniFrac, and fungal Jaccard indices (Figures 7, 8). PERMANOVA analysis was performed on physicochemical data to evaluate the correlation between these variables and the microbial composition. Titratable acidity was determined as a factor that was significant for bacterial Bray–Curtis dissimilarity (pseudo *F* = 7.1182, *R*^2^ = 0.20863, *p* = 0.001998) and weighted UniFrac (pseudo *F* = 13.242, *R*^2^ = 0.32906, *p* = 0.000999). The fungal community measured by Jaccard distance was determined to be significantly associated with water activity (pseudo *F* = 2.6309, *R*^2^ = 0.07823, *p* = 0.01199) and color (L*: *p* = 0.03497, a*: *p* = 0.007992, b*: *p* = 0.005994).

**Figure 9:**
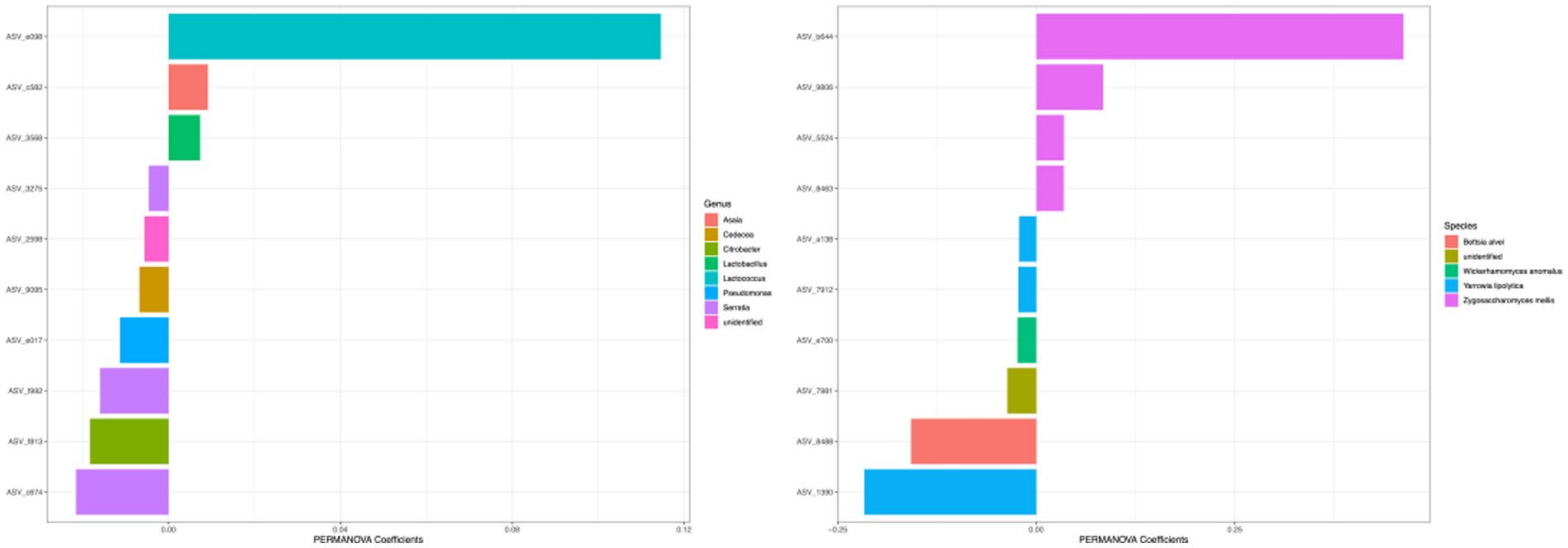
Top coefficient amplicon sequence variants (ASVs) for beta diversity. Top bacterial and fungal ASVs that were associated with community differences between samples as estimated by Bray–Curtis dissimilarity were plotted. Color of each bar represented the genera for 16S ASVs and species for ITS ASVs. The top 10 ASVs with the highest PERMANOVA coefficient values were plotted.

The differences in beta diversity of different types of honey can be attributed to the taxonomic composition of the microbiota, and the taxa with the highest coefficient values were visualized in Figure 9. For the bacterial community measured with Bray–Curtis metric, the top 5 ASVs with the largest effects on PERMANOVA coefficient were in the genera of *Lactococcus*, *Serratia*, *Citrobacter*, *Serratia*, and *Pseudomonas* (Two ASVs were identified as the same genus *Serratia*). For the fungal community, the top 5 ASVs with the largest effects on PERMANOVA coefficient were under the species of *Zygosaccharomyces mellis*, *Yarrowia lipolytica*, *Bettsia alvei*, *Zygosaccharomyces mellis*, and *Skoua* sp. (Two ASVs were identified as the same species *Zygosaccharomyces mellis*).

## 4. Discussion

Honey microbiota is a complex matrix that contains ecological information regarding the host microenvironment, the hive pathosphere, and the honeybee hologenome (Schwarz et al., 2015). Some bioindicators, including the agricultural and urban landscape, microbial environment that honeybees are exposed to, and the chemical pollutants in the foraging routes, can be reflected in the honey microbiota (Rissato et al., 2007; Lambert et al., 2012; Bargańska et al., 2016). NGS methods, including metabarcoding, can elucidate the complicated mutualism and symbiotic ecological relationships between honeybees and the environment (Bovo et al., 2018, 2020). The information we obtained from next-generation sequencing can provide taxonomic classification of honey microbiota and potentially be used as an indicator for the overall beehive health and honey origin (Bovo et al., 2020).

Most of the bacterial species identified in honey were osmotolerant, xerotolerant, and acidotolerant, considering that honey has a relatively high sugar content, low water activity, and low pH (Brudzynski, 2021). One of the most abundant bacterial species we found in our honey samples is *Lactococcus lactis*, which is consistently present in all honey samples we sequenced (Figure 1). *Lactococcus* is a member of the lactic acid bacteria (LAB), which are able to ferment carbohydrates in honey (fructose and glucose) and produce lactic acid. As a ubiquitous group of bacteria that are commonly found in plant materials, LAB have been isolated from honeybee hives and bee products in previous research (Sinacori et al., 2014; Kňazovická et al., 2020). Some secondary metabolites produced by LAB strains can inhibit spoilage organisms and pathogens and contribute to the overall beehive health. One example is *Lactobacillus kunkeei*, which is beneficial to the bee colony by protecting the hive from potential pathogens like *Paenibacillus larvae* and *Nosema ceranae* (Arredondo et al., 2018). As a ubiquitous species that is commonly found in flower, fruits, and soil, *L. kunkeei* is commonly associated with honeybee hive environment and bee products. *L. kunkeei* was found in honey bee bread using both culture-dependent and culture-independent method (Anderson et al., 2013). For honey samples in our study, *L. kunkeei* is one of the fructophiles that can be found in some but not all honey samples (Figure 1). Comparatively, another study on the microbiome of stingless bee honey revealed that the most abundant species is *Lactobacillus malefermentans*, and the top 7 OTUs in this study were all members of the genus *Lactobacillus* (Rosli et al., 2020). One of the possible reasons that *Lactobacillus* is missing in some of our honey samples is that *Lactobacillus* disappears below moisture content of 18% during honey ripening process (Ruiz-Argueso and Rodriguez-Navarro, 1975; Wen et al., 2017). Other studies also suggested that the presence of *L. kunkeei* is sporadic and its detection is dependent on the factors like floral source and season (Vásquez et al., 2012). Distinct differences can be seen when comparing our bacterial profile with the bacterial profile of vitex honey during ripening, which was dominated by *Bacillus* spp. (Wen et al., 2017). However, some bacteria with high abundance in vitex honey can be found in our honey samples, including *Lactococcus* and *Pseudomonas*. Some unresolved Enterobacteriaceae were present in our honey samples, which are likely from the pollination environment since they are frequently isolated from crops of forager bees (Corby-Harris et al., 2014). Even though gut microbiota could be a source of microbial community members in honey, many gut bacteria are considered gut-specific and do not survive well in other environments. Only *L. kunkeei* and *Acetobacteraceae* (*Asaia* spp.) were found in extreme conditions like honey and royal jelly (Martinson V. G. et al., 2012; Anderson et al., 2013; Vojvodic et al., 2013). *Serratia* is one of the most abundant genera found in our honey samples, which is consistent with a previous microbial metabarcoding study on three polyfloral honeys from Italy, where *Serratia symbiotica* was the fourth most abundant bacteria accounting for 4.8% of the bacteria reads (Bovo et al., 2020). *Serratia* is a common genus found in many insects (Dillon and Dillon, 2004). The origin of *Serratia* in honey is intriguing, since it is commonly associated with aphids as a secondary endosymbiont. It is possible that *Serratia* originated from honeydew produced by aphids, which was then fed to honeybees to produce honey (Bovo et al., 2020). Certain *Serratia* spp. have been characterized as opportunistic pathogens for honeybees, which could have an influence on the overall beehive health (Raymann et al., 2018).

For the fungal communities, diverse profiles can be observed across different types of honey. The most abundant fungal genera in our honey samples were *Bettsia*, *Yarrowia*, *Skoua*, *Zygosaccharomyces*, and *Metschnikowia*. Similar to our study, the fungal profile of vitex honey is also heterogeneous, with *Waitea*, *Phoma*, *Metschnikowia*, and *Cryptococcus* being the most predominant genera. *Metschnikowia* was found to be relatively stable in mature vitex honey and dominant in vitex flower. We propose that *Metschnikowia* and other yeasts in our honey samples originated from nectar, which can be transmitted from flower and fruits to honeybee products (Hong et al., 2001; Lievens et al., 2015). The absence of *Waitea* and *Cryptococcus* in our honey samples could be due to flower origin, since these two genera were found to be dominant in vitex flower (Wen et al., 2017). Culture-based methods identified yeasts like *Zygosaccharomyces* and *Debaryomyces* as the most prevalent genera in honey (Sinacori et al., 2014). In a culture-independent study with ITS2 metabarcoding, *Zygosaccharomyces* was the only species shared among almost all honey samples (Balzan et al., 2020). Filamentous fungi like *Aspergillus* are considered environmental contaminants for honey (Kacániová et al., 2009). A shotgun metagenomic study found that the second most represented fungus in polyfloral Italian honey was *Aspergillus flavus* (Bovo et al., 2020). *Aspergillus flavus* is a potential honeybee pathogen that could cause stonebrood disease, and was found to be abundant in some of our monofloral, wildflower, and manuka honey samples (Figure 2). Similarly, *Ascosphaera apis* is the causative agent for chalkbrood disease (Vojvodic et al., 2011). *Ascosphaera* sp. was found to be prevalent in some of the wildflower honey samples in our study (Figure 2). However, the presence of pathogenic fungi does not necessarily mean that the beehives are infected. Indeed, as shown in the study by Bovo et al. (2020), none of the sampled colonies that contained pathogenic fungi DNA in metagenomic analysis displayed any of these symptoms over 2 years. The onset of these diseases requires specific environment factors, and most of the pathogenic fungi only survive in honey as dormant spores.

In our study, we chose to not perform culture-based isolation methods due to culture biases. Performing bacterial and fungal culture isolation could not give us a whole picture of the microbiota, nor could it provide proof of the absence of certain species. As previous studies shown, species from genera *Bacillus* and *Paenibacillus* were considered dominant when evaluating the honey bacterial composition with culture-based method because aerobic plate counts were usually dominated by fast-growing bacteria like *Bacillus* spp., *Staphylococcus* spp. and *Paenibacillus* spp., while the dominant bacteria identified using amplicon sequencing were under-represented in culture-based methods due to various factors, like injured cells, persister cells, improper culture environment, or failing to compete with other organisms in culture (Iurlina and Fritz, 2005; Sinacori et al., 2014; Balzan et al., 2020). Moreover, plate count methods overestimated the bacteria abundance in honeybee stomach by over one order of magnitude, and core crop bacteria identified using culture-based method were inconsistent and occurred at low frequency when using qRT-PCR or NGS methods (Corby-Harris et al., 2014). In our opinion, using culture-independent methods to investigate the microbiome of honey avoids the growth condition and culture biases, and culture-based methods should not be performed as a complement to culture-independent amplicon sequencing or metagenomic studies. Alternatively, designing strain-specific primers and performing quantitative real-time PCR is the proper way to confirm the presence/absence of certain species identified by amplicon sequencing.

The physicochemical properties of different honey types in this study were highly comparable, especially for pH and Brix (Table 1). Color is one of the parameters that can be used to distinguish different honeys. Ecological diversity indices can be assessed based on the ASVs in different honeys, and the following hypotheses can be drawn by associating these diversity indices with honey physicochemical properties using appropriate statistical methods. In our study, titratable acidity was found to be correlated with bacterial alpha diversity metrics, including ACE richness, Shannon diversity, and inverse Simpson diversity. A few physicochemical factors were also found to be correlated with fungal Chao richness metric, including TA, Brix, and color. Furthermore, based on beta diversity metric correlation analysis, we determined that TA was a significant factor associated with the differences in bacterial communities, while water activity and color were associated with the differences in fungal communities. Previous studies showed that honey pH and acidity were independent of geographic origins but associated with nectar composition and botanical source (da Silva et al., 2016; Scholz et al., 2020). Honey age, moisture, and purchase source were considered as relevant factors for the microbial community in raw honey, while botanical origin only affected the fungal composition (Balzan et al., 2020). pH, water activity, and country of origin were considered as minor factors. In our study, moisture was not a significant factor shaping the bacterial or fungal community. Conversely, several previous studies showed that honey microbial profile was associated with its moisture. In the study by Wen et al. (2017), the fungal community of vitex honey was correlated with moisture. Honey with high moisture content is more likely to ferment and spoil. However, the moisture content variation in our honey samples was relatively small, which may be the reason that the moisture content was not a significant factor influencing the microbial community of our honey samples. Another group of researchers evaluated physicochemical parameters including pH, water content, free acidity, and electrical conductivity and determined that only electrical conductivity was associated with bacterial community of honey based on RDA analysis and permutation test (Kňazovická et al., 2020). In the study by Rosli et al. (2020), the authors considered that the microbiome of stingless bee honey was associated with physicochemical factors including pH, acidity, and moisture content. The marginal effects of limited sample size may contribute to the discrepancy among different studies. Some other authors also mentioned the geographic region may be an important factor influencing the microbial community in honeybee products (Disayathanoowat et al., 2020). We only included two geographic regions in our study, which is why we cannot draw any conclusions on its effect on the microbial community. Future metagenomic studies should take geographic location into consideration when evaluating factors that may impact the microbial community of honey. To fully understand the effects of geographic location and other relevant variables on the microbiome diversity, samples collected in different regions of US or world need to be included, with specific details on the geographic distribution, local flowering plants diversity, and the honeybee genetic background.

Next-generation sequencing tools provide a higher level of resolution of the community composition compared to traditional culture methods. Species with low abundance can be detected with in-depth sequencing, which enables us to evaluate the microbiome composition more precisely (Claesson et al., 2010; Gupta et al., 2019). Some studies have been performed to evaluate the floral source of honey using DNA metabarcoding for authentication, and the digestive tract microbiome of honeybees with metagenomic tools (de Vere et al., 2017; Graystock et al., 2017; Jones et al., 2018; Utzeri et al., 2018; Yun et al., 2018). Many of these studies used 16S rRNA amplicon sequencing, which is what we chose to use in our study to evaluate the composition of bacterial community in honey. Metabarcoding methods have high coverage, high sequencing depth, and are non-selective (Cao et al., 2017). However, the disadvantage is that most of the sequences are assigned to the taxon with high abundance, which may neglect some of the less common species in a community with high complexity (Clooney et al., 2016). The bacterial classification is also identified at genus level or above (Claesson et al., 2010). To avoid using a pre-defined percentage threshold to determine variants, we chose to use amplicon sequence variants (ASVs), which considers amplicon abundance and error rates to discard spurious sequences and retain biologically meaningful sequences (Callahan et al., 2017). This method has a finer resolution and identifies microorganisms at phenotypical levels (Rognes et al., 2016). Using ASVs to represent original sequences is considered a step forward compared to previous studies using operational taxonomic units (OTUs) to construct consensus sequences with 97% similarity, which inevitably loses some taxonomic information (Strube, 2021). In our study, we chose the 16S V3-V4 and 5.8S-ITS2 regions considering the limited read length of Illumina MiSeq. The potential sequencing biases from Illumina MiSeq is also the reason we condensed ASVs to the genus level for 16S amplicons and species level for ITS amplicons instead of using ASVs as individual units (Strube, 2021). Future studies should use the full 16S V1-V9 region and full-length ITS1-5.8S-ITS2 region to get better resolution of the honey bacterial and fungal population. Using sequencing platforms with higher read length and choosing proper primers for multiple barcode sequences will yield results with higher resolution.

This study contributes to the knowledge of environmental effects on microbial biodiversity and ecosystem associated with different types of honey. Investigation on the microbiome of honey and other bee products could shed light into honeybee diseases that causes significant ecological and economic damage, such as Colony Collapse Disorder (CCD; Cox-Foster et al., 2007). By comparing the microbiome of honey produced by different bee colonies, we can investigate the correlation between the microbiome and these honeybee diseases. Even though the presence of pathogenic microbial DNA may not directly correlate to honeybee diseases, using metagenomic tools to determine the relative abundance of these pathogens can provide information on possible hive diseases and overall beehive health. Future studies should focus on the shift of certain bacterial and fungal species in honey and other bee products to decipher the implication of honey microbiome on honeybee diseases and overall bee colony health.

## Data availability statement

The datasets presented in this study can be found in online repositories. The names of the repository/repositories and accession number(s) can be found in the article/Supplementary material.

## Author contributions

ZX contributed to data collection, data analysis, results interpretation, and writing—original draft. All authors contributed to the article and approved the submitted version.

## Funding

This study was supported by the U.S. Department of Agriculture, National Institute of Food and Agriculture multistate project S-1077, and the College of Agriculture and Life Sciences at Cornell University.

## Conflict of interest

The authors declare that the research was conducted in the absence of any commercial or financial relationships that could be construed as a potential conflict of interest.

## Publisher’s note

All claims expressed in this article are solely those of the authors and do not necessarily represent those of their affiliated organizations, or those of the publisher, the editors and the reviewers. Any product that may be evaluated in this article, or claim that may be made by its manufacturer, is not guaranteed or endorsed by the publisher.

## References

[ref1] Álvarez-Pérez S. Herrera C. M. de Vega C. (2012). Zooming-in on floral nectar: a first exploration of nectar-associated bacteria in wild plant communities. FEMS Microbiol. Ecol. 80, 591–602. doi: 10.1111/j.1574-6941.2012.01329.x, PMID: 22324904

[ref2] Alvarez-Suarez J. M. Gasparrini M. Forbes-Hernández T. Y. Mazzoni L. Giampieri F. (2014). The composition and biological activity of honey: a focus on Manuka honey. Foods 3, 420–432. doi: 10.3390/foods3030420, PMID: 28234328 PMC5302252

[ref3] Anderson K. E. Sheehan T. H. Mott B. M. Maes P. Snyder L. Schwan M. R.. (2013). Microbial ecology of the hive and pollination landscape: bacterial associates from floral nectar, the alimentary tract and stored food of honey bees (*Apis mellifera*). PLoS One 8:e83125. doi: 10.1371/journal.pone.0083125, PMID: 24358254 PMC3866269

[ref4] Arredondo D. Castelli L. Porrini M. P. Garrido P. M. Eguaras M. J. Zunino P.. (2018). *Lactobacillus kunkeei* strains decreased the infection by honey bee pathogens *Paenibacillus larvae* and *Nosema ceranae*. Benefic. Microbes 9, 279–290. doi: 10.3920/bm2017.0075, PMID: 29264966

[ref5] Balzan S. Carraro L. Merlanti R. Lucatello L. Capolongo F. Fontana F.. (2020). Microbial metabarcoding highlights different bacterial and fungal populations in honey samples from local beekeepers and market in North-Eastern Italy. Int. J. Food Microbiol. 334:108806. doi: 10.1016/j.ijfoodmicro.2020.108806, PMID: 32805512

[ref6] Bargańska Ż. Ślebioda M. Namieśnik J. (2016). Honey bees and their products: bioindicators of environmental contamination. Crit. Rev. Environ. Sci. Technol. 46, 235–248. doi: 10.1080/10643389.2015.1078220

[ref7] Barke J. Seipke R. F. Grüschow S. Heavens D. Drou N. Bibb M. J.. (2010). A mixed community of actinomycetes produce multiple antibiotics for the fungus farming ant *Acromyrmex octospinosus*. BMC Biol. 8:109. doi: 10.1186/1741-7007-8-109, PMID: 20796277 PMC2942817

[ref8] Beals E. W. (1984). “Bray-Curtis ordination: an effective strategy for analysis of multivariate ecological data” in Advances in Ecological Research. eds. MacFadyen A. Ford E. D. (London: Academic Press), 1–55.

[ref9] Bensch K. Braun U. Groenewald J. Z. Crous P. W. (2012). The genus Cladosporium. Stud. Mycol. 72, 1–401. doi: 10.3114/sim0003, PMID: 22815589 PMC3390897

[ref10] Bokulich N. A. Kaehler B. D. Rideout J. R. Dillon M. Bolyen E. Knight R.. (2018). Optimizing taxonomic classification of marker-gene amplicon sequences with QIIME 2’s q2-feature-classifier plugin. Microbiome 6:90. doi: 10.1186/s40168-018-0470-z, PMID: 29773078 PMC5956843

[ref11] Bolyen E. Rideout J. R. Dillon M. R. Bokulich N. A. Abnet C. C. Al-Ghalith G. A.. (2019). Reproducible, interactive, scalable and extensible microbiome data science using QIIME 2. Nat. Biotechnol. 37, 852–857. doi: 10.1038/s41587-019-0209-9, PMID: 31341288 PMC7015180

[ref12] Bovo S. Ribani A. Utzeri V. J. Schiavo G. Bertolini F. Fontanesi L. (2018). Shotgun metagenomics of honey DNA: evaluation of a methodological approach to describe a multi-kingdom honey bee derived environmental DNA signature. PLoS One 13:e0205575. doi: 10.1371/journal.pone.0205575, PMID: 30379893 PMC6209200

[ref13] Bovo S. Utzeri V. J. Ribani A. Cabbri R. Fontanesi L. (2020). Shotgun sequencing of honey DNA can describe honey bee derived environmental signatures and the honey bee hologenome complexity. Sci. Rep. 10:9279. doi: 10.1038/s41598-020-66127-1, PMID: 32518251 PMC7283317

[ref14] Brudzynski K. (2021). Honey as an ecological reservoir of antibacterial compounds produced by antagonistic microbial interactions in plant nectars, honey and honey bee. Antibiotics 10:551. doi: 10.3390/antibiotics10050551, PMID: 34065141 PMC8151657

[ref15] Brudzynski K. Abubaker K. St-Martin L. Castle A. (2011). Re-examining the role of hydrogen peroxide in bacteriostatic and bactericidal activities of honey. Front. Microbiol. 2:213. doi: 10.3389/fmicb.2011.00213, PMID: 22046173 PMC3201021

[ref16] Callahan B. J. McMurdie P. J. Holmes S. P. (2017). Exact sequence variants should replace operational taxonomic units in marker-gene data analysis. ISME J. 11, 2639–2643. doi: 10.1038/ismej.2017.119, PMID: 28731476 PMC5702726

[ref17] Callahan B. J. McMurdie P. J. Rosen M. J. Han A. W. Johnson A. J. Holmes S. P. (2016). DADA2: high-resolution sample inference from Illumina amplicon data. Nat. Methods 13, 581–583. doi: 10.1038/nmeth.3869, PMID: 27214047 PMC4927377

[ref18] Cao Y. Fanning S. Proos S. Jordan K. Srikumar S. (2017). A review on the applications of next generation sequencing technologies as applied to food-related microbiome studies. Front. Microbiol. 8:1829. doi: 10.3389/fmicb.2017.01829, PMID: 29033905 PMC5627019

[ref19] Chen C. Campbell L. Blair S. Carter D. (2012). The effect of standard heat and filtration processing procedures on antimicrobial activity and hydrogen peroxide levels in honey. Front. Microbiol. 3:265. doi: 10.3389/fmicb.2012.00265, PMID: 22866051 PMC3406342

[ref20] Claesson M. J. Wang Q. O’Sullivan O. Greene-Diniz R. Cole J. R. Ross R. P.. (2010). Comparison of two next-generation sequencing technologies for resolving highly complex microbiota composition using tandem variable 16S rRNA gene regions. Nucleic Acids Res. 38:e200. doi: 10.1093/nar/gkq873, PMID: 20880993 PMC3001100

[ref21] Clooney A. G. Fouhy F. Sleator R. D. Driscoll O. A. Stanton C. Cotter P. D.. (2016). Comparing apples and oranges?: next generation sequencing and its impact on microbiome analysis. PLoS One 11:e0148028. doi: 10.1371/journal.pone.0148028, PMID: 26849217 PMC4746063

[ref22] Corby-Harris V. Maes P. Anderson K. E. (2014). The bacterial communities associated with honey bee (*Apis mellifera*) foragers. PLoS One 9:e95056. doi: 10.1371/journal.pone.0095056, PMID: 24740297 PMC3989306

[ref23] Cox-Foster D. L. Conlan S. Holmes E. C. Palacios G. Evans J. D. Moran N. A.. (2007). A metagenomic survey of microbes in honey bee colony collapse disorder. Science 318, 283–287. doi: 10.1126/science.1146498, PMID: 17823314

[ref24] da Silva P. M. Gauche C. Gonzaga L. V. Costa A. C. O. Fett R. (2016). Honey: chemical composition, stability and authenticity. Food Chem. 196, 309–323. doi: 10.1016/j.foodchem.2015.09.051, PMID: 26593496

[ref25] Davis N. M. Proctor D. M. Holmes S. P. Relman D. A. Callahan B. J. (2018). Simple statistical identification and removal of contaminant sequences in marker-gene and metagenomics data. Microbiome 6:226. doi: 10.1186/s40168-018-0605-2, PMID: 30558668 PMC6298009

[ref26] de Vere N. Jones L. E. Gilmore T. Moscrop J. Lowe A. Smith D.. (2017). Using DNA metabarcoding to investigate honey bee foraging reveals limited flower use despite high floral availability. Sci. Rep. 7:42838. doi: 10.1038/srep42838, PMID: 28205632 PMC5311969

[ref27] Dillon R. J. Dillon V. (2004). The gut bacteria of insects: nonpathogenic interactions. Annu. Rev. Entomol. 49, 71–92. doi: 10.1146/annurev.ento.49.061802.123416 14651457

[ref28] Disayathanoowat T. Li H. Supapimon N. Suwannarach N. Lumyong S. Chantawannakul P.. (2020). Different dynamics of bacterial and fungal communities in hive-stored bee bread and their possible roles: a case study from two commercial honey bees in China. Microorganisms 8:264. doi: 10.3390/microorganisms8020264, PMID: 32075309 PMC7074699

[ref29] Engel P. Kwong W. K. McFrederick Q. Anderson K. E. Barribeau S. M. Chandler J. A.. (2016). The bee microbiome: impact on bee health and model for evolution and ecology of host-microbe interactions. MBio 7:e02164-02115. doi: 10.1128/mBio.02164-15 27118586 PMC4850275

[ref30] Engel P. Martinson V. G. Moran N. A. (2012). Functional diversity within the simple gut microbiota of the honey bee. Proc. Natl. Acad. Sci. U. S. A. 109, 11002–11007. doi: 10.1073/pnas.1202970109, PMID: 22711827 PMC3390884

[ref31] Fadrosh D. W. Ma B. Gajer P. Sengamalay N. Ott S. Brotman R. M.. (2014). An improved dual-indexing approach for multiplexed 16S rRNA gene sequencing on the Illumina MiSeq platform. Microbiome 2:6. doi: 10.1186/2049-2618-2-6, PMID: 24558975 PMC3940169

[ref32] Fridman S. Izhaki I. Gerchman Y. Halpern M. (2012). Bacterial communities in floral nectar. Environ. Microbiol. Rep. 4, 97–104. doi: 10.1111/j.1758-2229.2011.00309.x 23757235

[ref33] Genersch E. (2010). American foulbrood in honeybees and its causative agent, *Paenibacillus larvae*. J. Invertebr. Pathol. 103, S10–S19. doi: 10.1016/j.jip.2009.06.015 19909971

[ref34] Gilliam M. Wickerham L. J. Morton H. L. Martin R. D. (1974). Yeasts isolated from honey bees, Apis mellifera, fed 2,4-D and antibiotics. J. Invertebr. Pathol. 24, 349–356. doi: 10.1016/0022-2011(74)90143-8, PMID: 4443608

[ref35] Graystock P. Rehan S. M. McFrederick Q. S. (2017). Hunting for healthy microbiomes: determining the core microbiomes of Ceratina, Megalopta, and Apis bees and how they associate with microbes in bee collected pollen. Conserv. Genet. 18, 701–711. doi: 10.1007/s10592-017-0937-7

[ref36] Gupta S. Mortensen M. S. Schjørring S. Trivedi U. Vestergaard G. Stokholm J.. (2019). Amplicon sequencing provides more accurate microbiome information in healthy children compared to culturing. Commun. Biol. 2:291. doi: 10.1038/s42003-019-0540-1, PMID: 31396571 PMC6683184

[ref37] Hamdi C. Balloi A. Essanaa J. Crotti E. Gonella E. Raddadi N.. (2011). Gut microbiome dysbiosis and honeybee health. J. Appl. Entomol. 135, 524–533. doi: 10.1111/j.1439-0418.2010.01609.x

[ref38] Herlemann D. P. Labrenz M. Jürgens K. Bertilsson S. Waniek J. J. Andersson A. F. (2011). Transitions in bacterial communities along the 2000 km salinity gradient of the Baltic Sea. ISME J. 5, 1571–1579. doi: 10.1038/ismej.2011.41, PMID: 21472016 PMC3176514

[ref39] Hinshaw C. Evans K. C. Rosa C. López-Uribe M. M. (2021). The role of pathogen dynamics and immune gene expression in the survival of feral honey bees. Front. Ecol. Evol. 8:594263. doi: 10.3389/fevo.2020.594263

[ref40] Holm J. B. Humphrys M. S. Robinson C. K. Settles M. L. Ott S. Fu L.. (2019). Ultrahigh-throughput multiplexing and sequencing of 500-base-pair amplicon regions on the Illumina HiSeq 2500 platform. mSystems 4:e00029-00019. doi: 10.1128/mSystems.00029-19, PMID: 30801027 PMC6381223

[ref41] Hong S. G. Chun J. Oh H. W. Bae K. S. (2001). Metschnikowia koreensis sp. nov., a novel yeast species isolated from flowers in Korea. Int. J. Syst. Evol. Microbiol. 51, 1927–1931. doi: 10.1099/00207713-51-5-1927 11594627

[ref42] Iurlina M. O. Fritz R. (2005). Characterization of microorganisms in Argentinean honeys from different sources. Int. J. Food Microbiol. 105, 297–304. doi: 10.1016/j.ijfoodmicro.2005.03.017, PMID: 16169624

[ref43] Jaccard P. (1912). The distribution of the flora in the alpine zone. 1. New Phytol. 11, 37–50. doi: 10.1111/j.1469-8137.1912.tb05611.x

[ref44] Jensen A. B. Aronstein K. Flores J. M. Vojvodic S. Palacio M. A. Spivak M. (2013). Standard methods for fungal brood disease research. J. Apic. Res. 52, 1–20. doi: 10.3896/IBRA.1.52.1.13, PMID: 24198438 PMC3816652

[ref45] Jones J. C. Fruciano C. Hildebrand F. Al Toufalilia H. Balfour N. J. Bork P.. (2018). Gut microbiota composition is associated with environmental landscape in honey bees. Ecol. Evol. 8, 441–451. doi: 10.1002/ece3.3597, PMID: 29321884 PMC5756847

[ref46] Kacániová M. Pavlicová S. Hascík P. Kociubinski G. Kńazovická V. Sudzina M.. (2009). Microbial communities in bees, pollen and honey from Slovakia. Acta Microbiol. Immunol. Hung. 56, 285–295. doi: 10.1556/AMicr.56.2009.3.7, PMID: 19789142

[ref47] Klindworth A. Pruesse E. Schweer T. Peplies J. Quast C. Horn M.. (2013). Evaluation of general 16S ribosomal RNA gene PCR primers for classical and next-generation sequencing-based diversity studies. Nucleic Acids Res. 41:e1. doi: 10.1093/nar/gks808, PMID: 22933715 PMC3592464

[ref48] Kňazovická V. Gábor M. Miluchová M. Bobko M. Medo J. (2020). Diversity of bacteria in Slovak and foreign honey, with assessment of its physico-chemical quality and counts of cultivable microorganisms. J. Microbiol. Biotechnol. Food Sci. 9, 414–421. doi: 10.15414/jmbfs.2019.9.special.414-421

[ref49] Kurek-Górecka A. Górecki M. Rzepecka-Stojko A. Balwierz R. Stojko J. (2020). Bee products in dermatology and skin care. Molecules 25:556. doi: 10.3390/molecules25030556, PMID: 32012913 PMC7036894

[ref50] Lambert O. Piroux M. Puyo S. Thorin C. Larhantec M. Delbac F.. (2012). Bees, honey and pollen as sentinels for lead environmental contamination. Environ. Pollut. 170, 254–259. doi: 10.1016/j.envpol.2012.07.012, PMID: 22842054

[ref51] Lievens B. Hallsworth J. E. Pozo M. I. Belgacem Z. B. Stevenson A. Willems K. A.. (2015). Microbiology of sugar-rich environments: diversity, ecology and system constraints. Environ. Microbiol. 17, 278–298. doi: 10.1111/1462-2920.12570, PMID: 25041632

[ref52] Lozupone C. A. Hamady M. Kelley S. T. Knight R. (2007). Quantitative and qualitative beta diversity measures lead to different insights into factors that structure microbial communities. Appl. Environ. Microbiol. 73, 1576–1585. doi: 10.1128/aem.01996-06, PMID: 17220268 PMC1828774

[ref53] Lozupone C. Knight R. (2005). UniFrac: a new phylogenetic method for comparing microbial communities. Appl. Environ. Microbiol. 71, 8228–8235. doi: 10.1128/aem.71.12.8228-8235.2005, PMID: 16332807 PMC1317376

[ref54] Lücking R. Aime M. C. Robbertse B. Miller A. N. Ariyawansa H. A. Aoki T.. (2020). Unambiguous identification of fungi: where do we stand and how accurate and precise is fungal DNA barcoding? IMA Fungus 11:14. doi: 10.1186/s43008-020-00033-z, PMID: 32714773 PMC7353689

[ref55] Martinson E. O. Herre E. A. Machado C. A. Arnold A. E. (2012). Culture-free survey reveals diverse and distinctive fungal communities associated with developing figs (*Ficus* spp.) in Panama. Microb. Ecol. 64, 1073–1084. doi: 10.1007/s00248-012-0079-x, PMID: 22729017

[ref56] Martinson V. G. Moy J. Moran N. A. (2012). Establishment of characteristic gut bacteria during development of the honeybee worker. Appl. Environ. Microbiol. 78, 2830–2840. doi: 10.1128/aem.07810-11, PMID: 22307297 PMC3318792

[ref57] McMurdie P. J. Holmes S. (2013). Phyloseq: an R package for reproducible interactive analysis and graphics of microbiome census data. PLoS One 8:e61217. doi: 10.1371/journal.pone.0061217, PMID: 23630581 PMC3632530

[ref58] Mohr K. I. Tebbe C. C. (2006). Diversity and phylotype consistency of bacteria in the guts of three bee species (Apoidea) at an oilseed rape field. Environ. Microbiol. 8, 258–272. doi: 10.1111/j.1462-2920.2005.00893.x, PMID: 16423014

[ref59] Moran N. A. Hansen A. K. Powell J. E. Sabree Z. L. (2012). Distinctive gut microbiota of honey bees assessed using deep sampling from individual worker bees. PLoS One 7:e36393. doi: 10.1371/journal.pone.0036393, PMID: 22558460 PMC3338667

[ref60] Niaz K. Maqbool F. Bahadar H. Abdollahi M. (2017). Health benefits of Manuka honey as an essential constituent for tissue regeneration. Curr. Drug Metab. 18, 881–892. doi: 10.2174/1389200218666170911152240, PMID: 28901255

[ref61] Olaitan P. B. Adeleke O. E. Ola I. O. (2007). Honey: a reservoir for microorganisms and an inhibitory agent for microbes. Afr. Health Sci. 7, 159–165. doi: 10.5555/afhs.2007.7.3.159, PMID: 18052870 PMC2269714

[ref62] Olofsson T. C. Vásquez A. (2008). Detection and identification of a novel lactic acid bacterial flora within the honey stomach of the honeybee *Apis mellifera*. Curr. Microbiol. 57, 356–363. doi: 10.1007/s00284-008-9202-0, PMID: 18663527

[ref63] Powell J. E. Martinson V. G. Urban-Mead K. Moran N. A. Goodrich-Blair H. (2014). Routes of acquisition of the gut microbiota of the honey bee *Apis mellifera*. Appl. Environ. Microbiol. 80, 7378–7387. doi: 10.1128/AEM.01861-14, PMID: 25239900 PMC4249178

[ref64] Raymann K. Coon K. L. Shaffer Z. Salisbury S. Moran N. A. (2018). Pathogenicity of *Serratia marcescens* strains in honey bees. MBio 9:e01649-01618. doi: 10.1128/mBio.01649-18 30301854 PMC6178626

[ref65] Raymann K. Moran N. A. (2018). The role of the gut microbiome in health and disease of adult honey bee workers. Curr. Opin. Insect Sci. 26, 97–104. doi: 10.1016/j.cois.2018.02.012, PMID: 29764668 PMC6010230

[ref66] Rissato S. R. Galhiane M. S. de Almeida M. V. Gerenutti M. Apon B. M. (2007). Multiresidue determination of pesticides in honey samples by gas chromatography–mass spectrometry and application in environmental contamination. Food Chem. 101, 1719–1726. doi: 10.1016/j.foodchem.2005.10.034

[ref67] Rivers A. Weber K. Gardner T. Liu S. Armstrong S. (2018). ITSxpress: software to rapidly trim internally transcribed spacer sequences with quality scores for marker gene analysis [version 1; peer review: 2 approved]. F1000Res. 7:1418. doi: 10.12688/f1000research.15704.1 30416717 PMC6206612

[ref68] Rognes T. Flouri T. Nichols B. Quince C. Mahé F. (2016). VSEARCH: a versatile open source tool for metagenomics. PeerJ 4:e2584. doi: 10.7717/peerj.2584, PMID: 27781170 PMC5075697

[ref69] Rosli F. N. Hazemi M. H. F. Akbar M. A. Basir S. Kassim H. Bunawan H. (2020). Stingless bee honey: evaluating its antibacterial activity and bacterial diversity. Insects 11:500. doi: 10.3390/insects11080500, PMID: 32759701 PMC7469184

[ref70] Ruiz-Argueso T. Rodriguez-Navarro A. (1975). Microbiology of ripening honey. Appl. Microbiol. 30, 893–896. doi: 10.1128/am.30.6.893-896.1975, PMID: 16350044 PMC376564

[ref71] Schoch C. L. Seifert K. A. Huhndorf S. Robert V. Spouge J. L. Levesque C. A.. (2012). Nuclear ribosomal internal transcribed spacer (ITS) region as a universal DNA barcode marker for fungi. Proc. Natl. Acad. Sci. U. S. A. 109, 6241–6246. doi: 10.1073/pnas.1117018109, PMID: 22454494 PMC3341068

[ref72] Scholz M. B. D. S. Quinhone Júnior A. Delamuta B. H. Nakamura J. M. Baudraz M. C. Reis M. O.. (2020). Indication of the geographical origin of honey using its physicochemical characteristics and multivariate analysis. J. Food Sci. Technol. 57, 1896–1903. doi: 10.1007/s13197-019-04225-3, PMID: 32327800 PMC7171034

[ref73] Schwarz R. S. Huang Q. Evans J. D. (2015). Hologenome theory and the honey bee pathosphere. Curr. Opin. Insect Sci. 10, 1–7. doi: 10.1016/j.cois.2015.04.006, PMID: 29587997

[ref74] Sinacori M. Francesca N. Alfonzo A. Cruciata M. Sannino C. Settanni L.. (2014). Cultivable microorganisms associated with honeys of different geographical and botanical origin. Food Microbiol. 38, 284–294. doi: 10.1016/j.fm.2013.07.013, PMID: 24290653

[ref75] Snowdon J. A. Cliver D. O. (1996). Microorganisms in honey. Int. J. Food Microbiol. 31, 1–26. doi: 10.1016/0168-1605(96)00970-1 8880294

[ref76] Strube M. L. (2021). RibDif: can individual species be differentiated by 16S sequencing? Bioinformatics Adv. 1. doi: 10.1093/bioadv/vbab020 PMC9710640 36700109

[ref77] Taylor D. L. Walters W. A. Lennon N. J. Bochicchio J. Krohn A. Caporaso J. G.. (2016). Accurate estimation of fungal diversity and abundance through improved lineage-specific primers optimized for Illumina amplicon sequencing. Appl. Environ. Microbiol. 82, 7217–7226. doi: 10.1128/AEM.02576-16, PMID: 27736792 PMC5118932

[ref78] Utzeri V. J. Ribani A. Schiavo G. Bertolini F. Bovo S. Fontanesi L. (2018). Application of next generation semiconductor based sequencing to detect the botanical composition of monofloral, polyfloral and honeydew honey. Food Control 86, 342–349. doi: 10.1016/j.foodcont.2017.11.033

[ref79] Vásquez A. Forsgren E. Fries I. Paxton R. J. Flaberg E. Szekely L.. (2012). Symbionts as major modulators of insect health: lactic acid bacteria and honeybees. PLoS One 7:e33188. doi: 10.1371/journal.pone.0033188, PMID: 22427985 PMC3299755

[ref80] Vojvodic S. Jensen A. B. James R. R. Boomsma J. J. Eilenberg J. (2011). Temperature dependent virulence of obligate and facultative fungal pathogens of honeybee brood. Vet. Microbiol. 149, 200–205. doi: 10.1016/j.vetmic.2010.10.001, PMID: 21050682

[ref81] Vojvodic S. Rehan S. M. Anderson K. E. (2013). Microbial gut diversity of Africanized and European honey bee larval instars. PLoS One 8:e72106. doi: 10.1371/journal.pone.0072106, PMID: 23991051 PMC3749107

[ref82] Wen Y. Wang L. Jin Y. Zhang J. Su L. Zhang X.. (2017). The microbial community dynamics during the vitex honey ripening process in the honeycomb. Front. Microbiol. 8:1649. doi: 10.3389/fmicb.2017.01649, PMID: 28912763 PMC5583594

[ref83] Youngsteadt E. Appler R. H. López-Uribe M. M. Tarpy D. R. Frank S. D. (2015). Urbanization increases pathogen pressure on feral and managed honey bees. PLoS One 10:e0142031. doi: 10.1371/journal.pone.0142031, PMID: 26536606 PMC4633120

[ref84] Yun J. H. Jung M. J. Kim P. S. Bae J. W. (2018). Social status shapes the bacterial and fungal gut communities of the honey bee. Sci. Rep. 8:2019. doi: 10.1038/s41598-018-19860-7, PMID: 29386588 PMC5792453

