# Supplementary material for: Microbiome analysis of raw honey reveals important factors influencing the bacterial and fungal communities
Source: Front Microbiol. 2023 Jan 12;13:1099522. doi: 10.3389/fmicb.2022.1099522 (PMC9877413; doi:10.3389/fmicb.2022.1099522)
Supplement: Supplementary file 1 [file Data_Sheet_1.pdf]

Supplementary Material

1 Supplementary Figures and Tables

1.1 Supplementary Figures

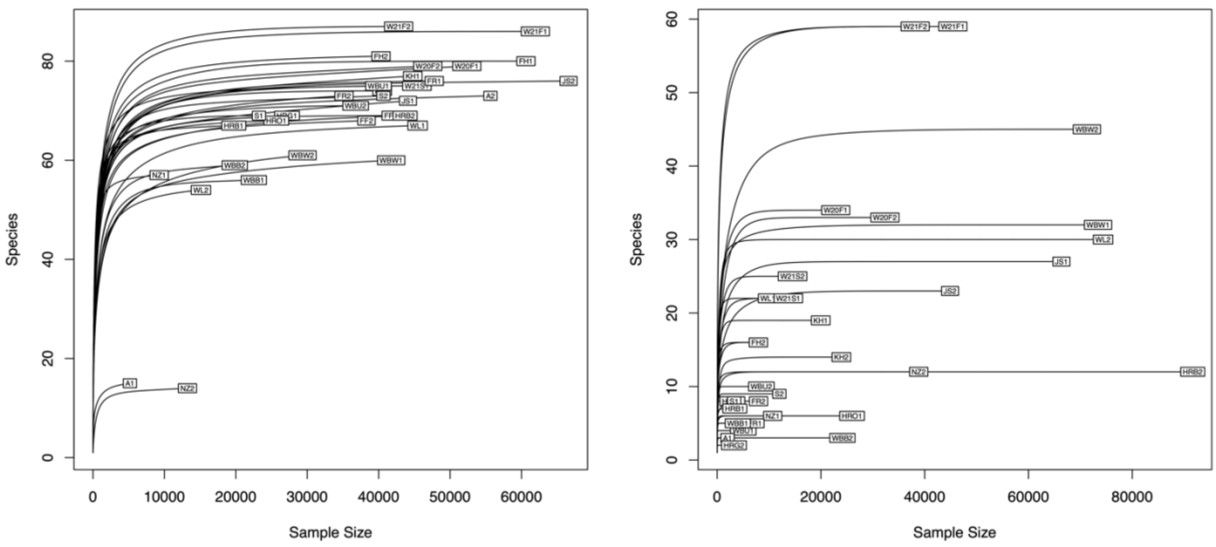

Supplementary Figure 1. Rarefaction curves of bacterial and fungal ASV diversity for each honey sample based on the number of obtained reads in the sequencing libraries and identified species.

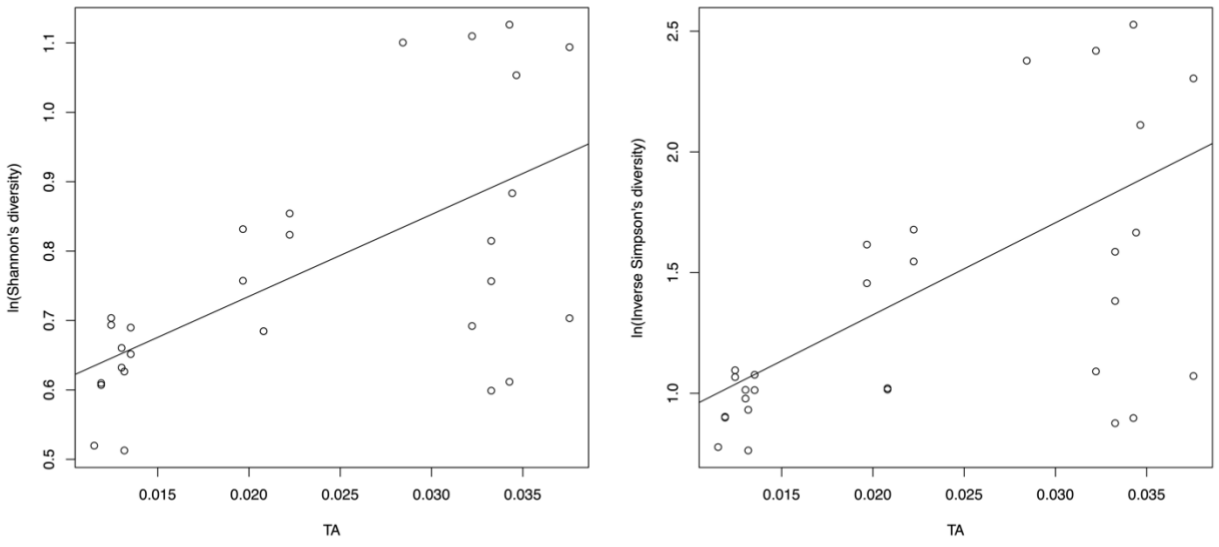

Supplementary Figure 2. Correlations between titratable acidity (TA) of honey samples and their bacterial community alpha diversity metrics. Shannon diversity and inverse Simpson diversity metrics were log-transformed and plotted with lines fitted by quasi-Poisson generalized linear model.

1.2 Supplementary Tables

Supplementary Table 1. Alpha diversity values for bacterial community of each honey sample based on 16S ASVs.

Sample ID	Honey Type	shannon	invsimp	chao1	ACE
FH1	Feral Honey	1.983	2.761	70.30	71.151
FH2	Feral Honey	1.983	2.776	77.60	78.856
FR1	Feral Honey	1.840	2.467	73.11	74.724
FR2	Feral Honey	1.835	2.458	65.00	65.161
A2	Manuka Honey	1.820	2.402	70.25	67.786
NZ1	Manuka Honey	3.006	10.779	58.00	58.498
S1	Manuka Honey	3.084	12.513	67.50	67.912
S2	Manuka Honey	1.843	2.453	70.33	71.621
HRB1	Monofloral Honey	3.033	11.231	65.50	66.304
HRB2	Monofloral Honey	1.998	2.976	59.67	60.286
HRG1	Monofloral Honey	2.419	5.289	70.60	69.393
HRO1	Monofloral Honey	2.867	8.259	68.00	66.454

WBB1	Monofloral Honey	2.132	4.288	56.00	57.328
WBB2	Monofloral Honey	2.297	5.033	57.20	57.280
WBU1	Monofloral Honey	2.986	10.015	71.50	71.779
WBU2	Monofloral Honey	2.020	2.920	66.60	67.114
WBW1	Monofloral Honey	2.131	3.984	54.00	52.033
WBW2	Monofloral Honey	2.258	4.885	60.00	59.319
WL1	Monofloral Honey	2.278	4.692	81.00	70.189
WL2	Monofloral Honey	2.349	5.355	51.20	51.837
JS1	Wildflower Honey	2.021	2.990	66.00	66.527
JS2	Wildflower Honey	2.001	2.907	70.67	71.946
KH1	Wildflower Honey	1.993	2.935	72.00	68.728
KH2	Wildflower Honey	1.919	2.754	69.50	70.880
W20F1	Wildflower Honey	1.882	2.659	74.50	75.074
W20F2	Wildflower Honey	1.936	2.756	69.00	70.395
W21F1	Wildflower Honey	1.871	2.538	84.88	84.474
W21F2	Wildflower Honey	1.670	2.145	86.75	85.277
W21S1	Wildflower Honey	1.681	2.175	76.00	71.887

Supplementary Table 2. Alpha diversity values for fungal community of each honey sample based on ITS ASVs.

Sample ID	Honey Type	shannon	invsimp	chao1	Evenness
FH1	Feral Honey	1.485	2.644	16.00	0.536
FH2	Feral Honey	1.768	3.478	16.00	0.638
FR1	Feral Honey	0.988	1.994	5.00	0.614
FR2	Feral Honey	1.031	1.951	8.00	0.496

A1	Manuka Honey	0.896	2.135	3.00	0.815
A2	Manuka Honey	0.582	1.365	5.00	0.362
NZ1	Manuka Honey	0.674	1.434	6.00	0.376
NZ2	Manuka Honey	1.246	2.394	12.00	0.501
S1	Manuka Honey	2.010	7.043	8.00	0.967
S2	Manuka Honey	1.071	2.039	9.00	0.487
HRB1	Monofloral Honey	1.597	4.366	7.00	0.821
HRB2	Monofloral Honey	1.135	2.548	11.00	0.473
HRG1	Monofloral Honey	1.769	4.518	8.00	0.851
HRG2	Monofloral Honey	0.268	1.162	2.00	0.386
HRO1	Monofloral Honey	1.068	2.445	6.00	0.596
WBB1	Monofloral Honey	1.150	2.385	5.00	0.715
WBB2	Monofloral Honey	0.743	2.000	3.00	0.676
WBU1	Monofloral Honey	0.969	2.198	4.00	0.699
WBU2	Monofloral Honey	1.040	1.764	10.00	0.452
WBW1	Monofloral Honey	1.011	1.589	32.00	0.297
WBW2	Monofloral Honey	1.087	1.742	41.25	0.320
WL1	Monofloral Honey	1.786	3.197	22.00	0.578
WL2	Monofloral Honey	1.248	1.944	29.75	0.371
JS1	Wildflower Honey	1.361	2.459	25.00	0.440
JS2	Wildflower Honey	1.432	2.642	33.00	0.496
KH1	Wildflower Honey	1.335	2.881	19.00	0.453
KH2	Wildflower Honey	1.226	2.730	13.00	0.478
W20F1	Wildflower Honey	1.854	4.890	35.67	0.540
W20F2	Wildflower Honey	1.828	4.828	33.00	0.538
W21F1	Wildflower Honey	2.633	7.358	54.00	0.673
W21F2	Wildflower Honey	2.518	6.199	52.50	0.647

W21S1	Wildflower Honey	0.701	1.320	26.00	0.234
W21S2	Wildflower Honey	1.292	2.253	23.00	0.412
